# Supplementary material for: Ebola Virus Encodes Two microRNAs in Huh7-Infected Cells
Source: Int J Mol Sci. 2022 May 7;23(9):5228. doi: 10.3390/ijms23095228 (PMC9106010; doi:10.3390/ijms23095228)
Supplement: Supplementary file 1 [file ijms-23-05228-s001.zip › Suppl_File_MS_Diallo_et_al_EBOLA_microRNAs.pdf]

International Journal of Molecular Sciences

MDPI

Research Article

Ebola virus encodes for two microRNAs in Huh7-infected cells

Idrissa Diallo[1], Zeinab Husseini[1], Sara Guellal[1], Elodie Vion[1], Jeffrey Ho[1], Robert Kozak[2,3], Gary Kobinger[1,2,4] and Patrick Provost[1]*

Supplementary Tables and Figures

Supplementary Table S1. Primer sequences used in RT-qPCR assays in Huh7 cells.

	Gene	5'-3' Sequence (Forward)<br>5'-3' Sequence (Reverse)	Amplicons (length; nt)	Tm (°C)	Accession number
1	DUSP16	GTGAAGAAAAGTTGCGAGCGT<br>ACTGGACTGAAAGCTTTTGGGG	269	60.51	NM_030640
2	NAMPT	GGAGCATCTGCTCACTTGGT<br>CCCCAAGCTGTTATGGTACTGT	143	60.04	NM_005746.3
3	FGD1	TCGTCTGGGACTACTGGCTG<br>CCAGGGTCAAGGGACAAACT	995	64.02	NM_004463.3
4	PUM2	TGTGTCTCACCCTCGGTACT<br>AAAAGCTCTCCCATTCCCCG	278	60	NM_015317.5
5	UNC5D	GGGTGCCCATTCAGCGG<br>TCGCCATTGTCAGTTCCTCG	298	60.62	NM_080872.4
6	PLCB4	CTCCAGGCAAAGTGACAGGAAA<br>TGCAGGTGATGTCCACTTCG	118	60.54	NM_000933.4
7	CDK13	AGCAGCTAACCTACAGTTGAAGA<br>AACAATTTCCCTGTCCTCCCA	165	59.32	NM_003718
8	VAPA	TGAAGACTACAGCACCTCGC<br>CCCTGGTGGAGTTATACCCAAT	284	59.49	NM_003574.6
9	P2RY13	CTGAACTAATGACTGCCGCC<br>GCTTCCAGTGTCACCTTTGG	70	59.02	NM_176894.3
10	WDR7	TCCGTAGTTCCGCCCTATCC<br>GAAGGCAACTGGCAGGTAGT	983	60	NM_015285.3
11	INPP5E	GGGAAGGACACGTACGACAG<br>GCTGCCAACGGAATGTTGTC	203	60.25	NM_019892.6
12	STAG2	GGGTGTAAAGAAGAGGAAGGGG<br>GAAAAATGTGTTTCTGACTCCTGTA	360	58.89	NM_001042749.2
13	SMURF2	CTGCGCCTGACAGGACTTC<br>TCATTGTCATTTGGCCCGAGT	299	60.51	NM_022739.4
14	CGRRF1	ACATCGCGGTGGTCTTTACC<br>ACATCCCAACCAAACCATCCC	70	60.47	NM_006568.3

The gene names listed from 1 to 6 are those targeted by EBOV-miR-MAY-251, and those from 7 to 14 by EBOV-miR-MAK-403. Tm, melting temperature; nt, nucleotides.

International Journal of *Molecular Sciences*

MDPI

Supplementary Table S2. Potential mRNA targets and the position of their binding sites for the two EBOV microRNAs, EBOV-miR-MAY-251 and EBOV-miR-MAK-403.

	**Gene**	**Minimum free energy (kcal/mol)**	**EBOV microRNA binding site position (nt)**
**1**	**DUSP16**	-25.6 (others are <= -23.9)	2024 (2150, 3023, 6574, 2665)
**2**	**NAMPT**	-24.2 (others are <= -21.5)	2986 (46, 577, 339, 458)
**3**	**FGD1**	-25.5 (others are <= -23.9)	3481 (1169, 4223, 837, 1517)
**4**	**PUM2**	-25.8 (others are <= -22.7)	1269 (1023, 1350, 1650, 1148)
**5**	**UNC5D**	-26.0 (others are <= -22.8)	2080 (3437, 1438, 5963, 7041)
**6**	**PLCB4**	-26.4 (others are <= -23.1)	1863 (659, 464, 3258, 4653)
**7**	**CDK13**	-22.6 (others are <= -21.0)	3927 (5461, 8631, 4925, 8484)
**8**	**VAPA**	-22.6 (others are <= -20.6)	5211 (1141, 4661, 1585, 1357)
**9**	**P2RY13**	-24.1 (others are <= -18.3)	364 (436, 1187, 2485, 2179)
**10**	**WDR7**	-24.6 (others are <= -19.7)	1166 (2733, 902, 5892, 4850)
**11**	**INPP5E**	-22.9 (others are <= -18.0)	3285 (2185, 1505, 1666, 2765)
**12**	**STAG2**	-22.4 (others are <= -21.2)	6179 (5405, 4360, 5977, 525)
**13**	**SMURF2**	-21.0 (others are <= -18.9)	5997 (2447, 4565, 710, 5568)
**14**	**CGRRF1**	-20.2 (others are <= -16.7)	1249 (684, 772, 235, 973)

RNAhybrid was used to calculate minimum free energy of each EBOV microRNA-mRNA interaction. The position (nt, nucleotide) for the highest interactions is shown in the table. The gene names listed from 1 to 6 are those targeted by EBOV-miR-MAY-251, and those from 7 to 14 by EBOV-miR-MAK-403.

International Journal of *Molecular Sciences*

MDPI

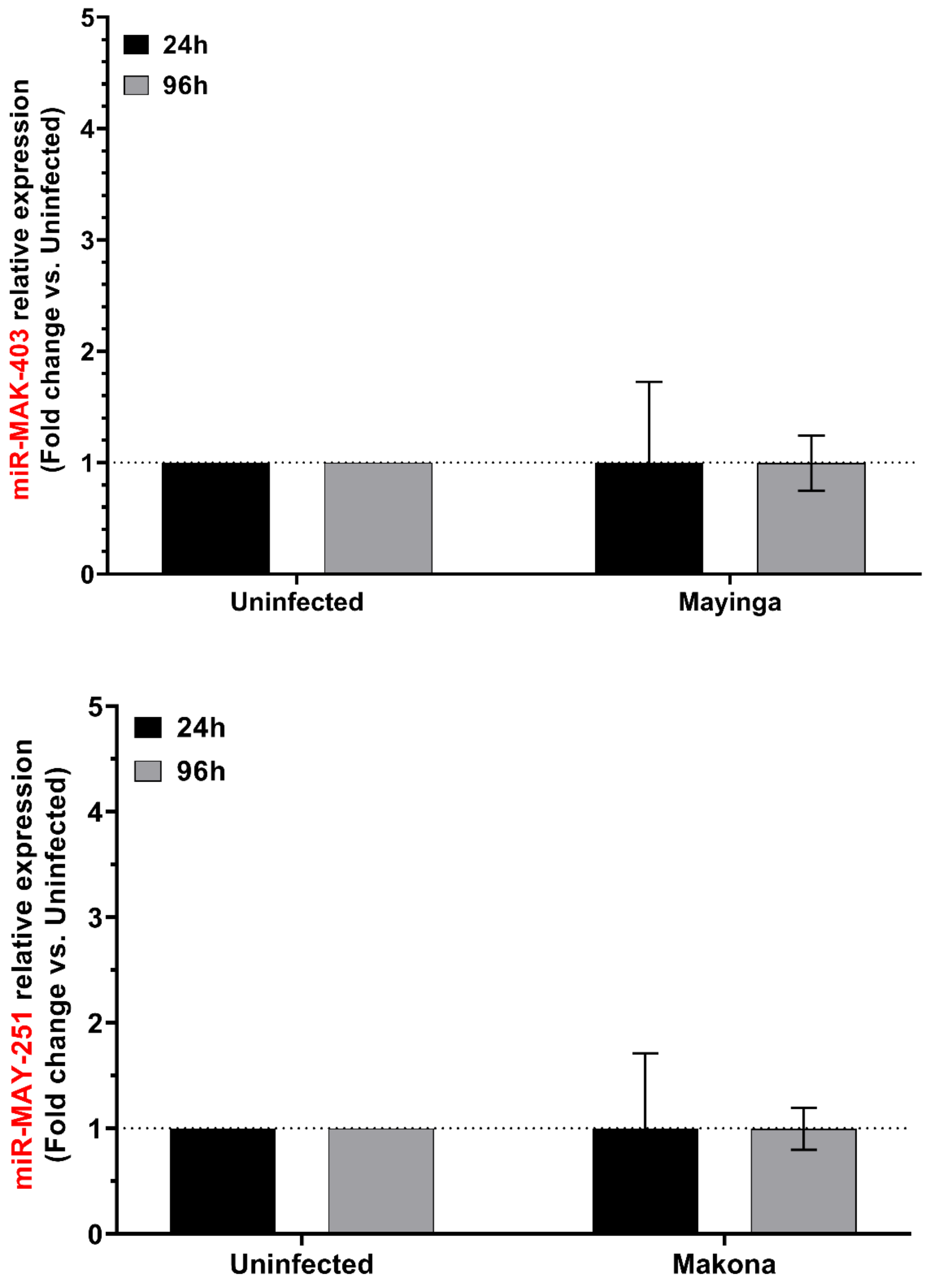

Supplementary Figure S1. Expression of EBOV-miR-MAY-251 and EBOV-miR-MAK-403 is specific to their viral genome of origin. RT-qPCR data showing relative expression of EBOV-miR-MAY-251 and EBOV-miR-MAK-403 in Huh7 cells infected for 24 h and 96 h with Makona or Mayinga EBOV strain, respectively. qPCR data were normalized with Unisp6 spike-in, reported to control (uninfected), and expressed with a relative quantitation method (ddCT). Data were calculated from three biological replicates and expressed as mean ± SD. **Statistical analysis**. Two-way analysis of variance (ANOVA) and Sidak multiple comparisons were used. No statistically significant difference (fold change vs uninfected) was observed.

International Journal of
Molecular Sciences

MDPI

A) miR-MAY-251

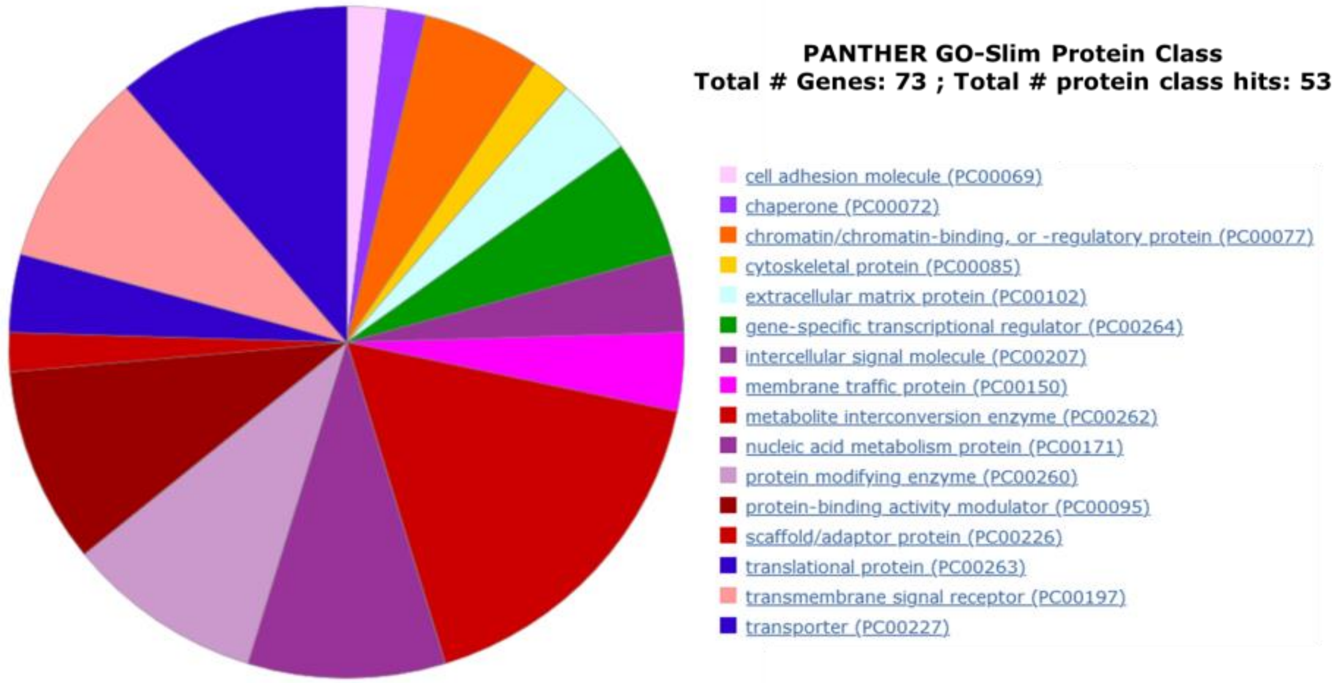

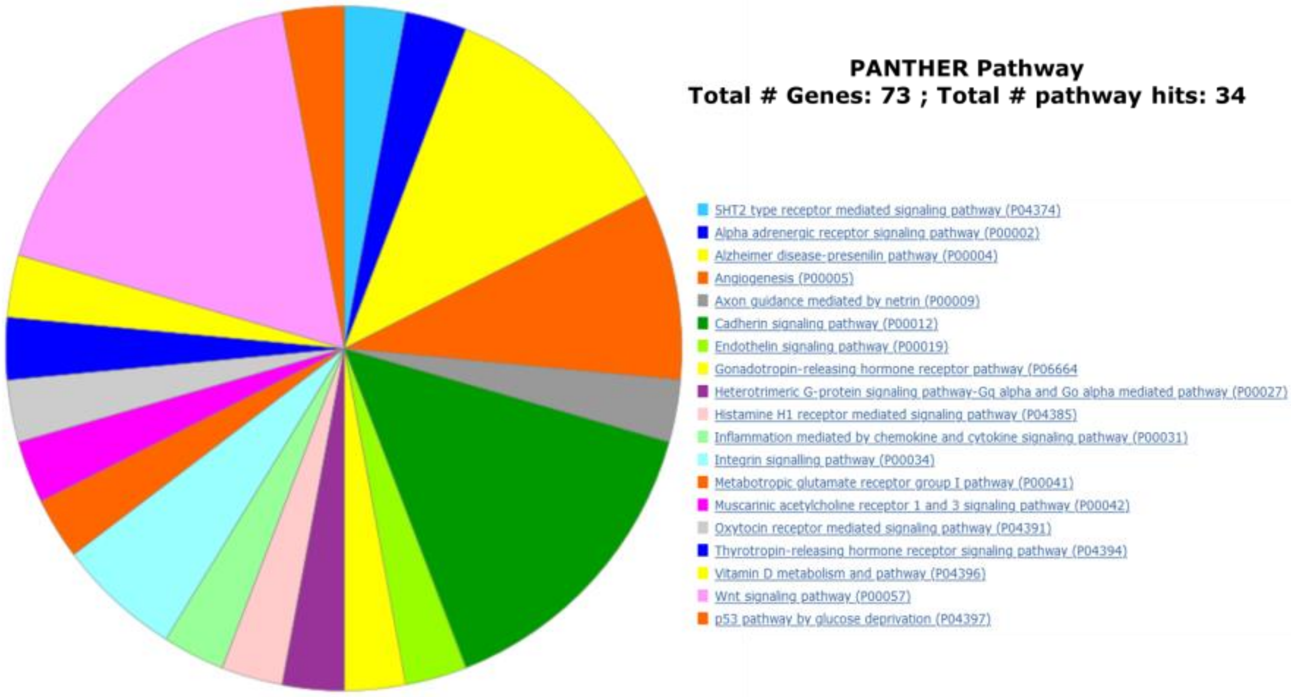

International Journal of
Molecular Sciences

MDPI

B) miR-MAK-403

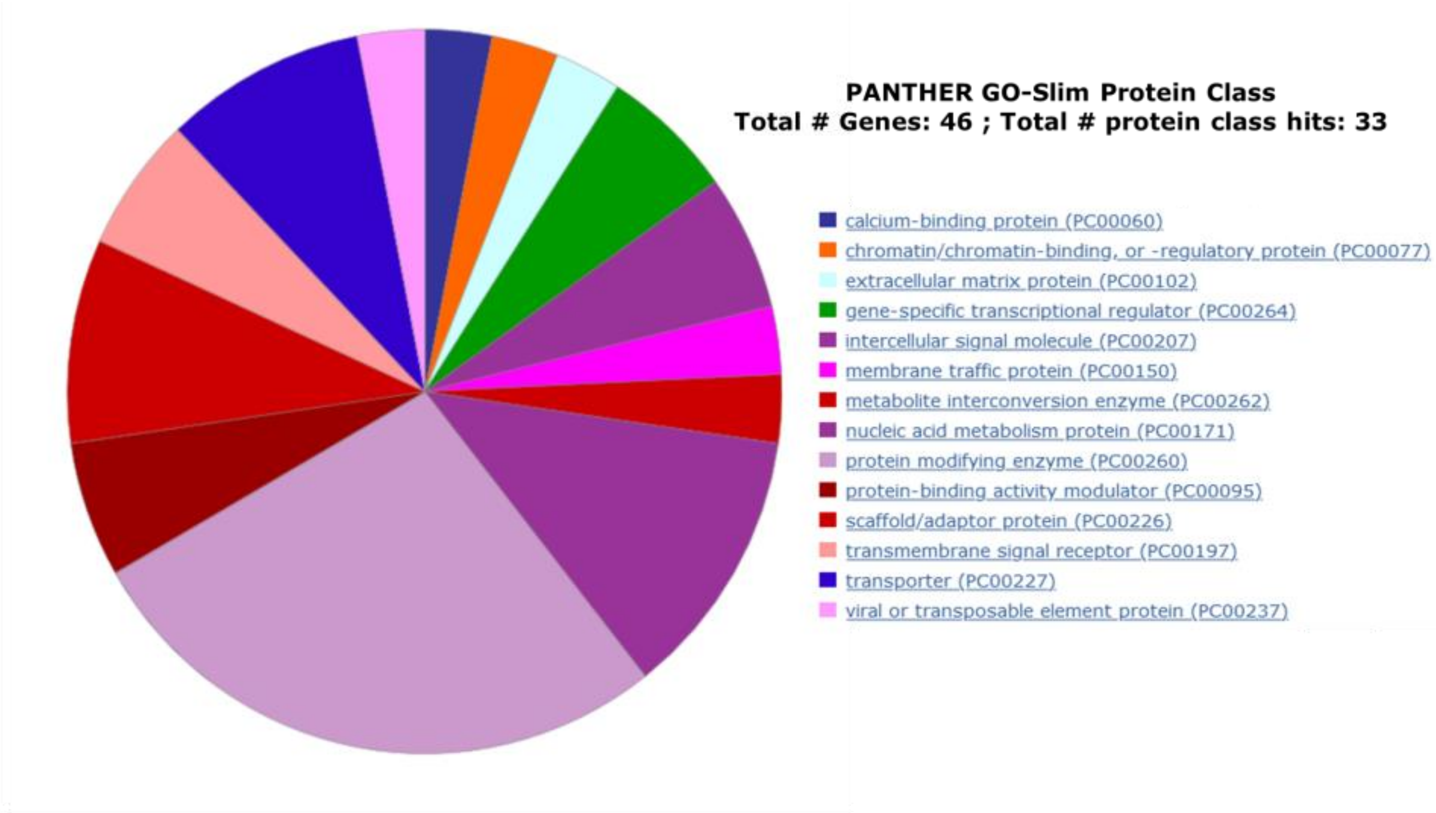

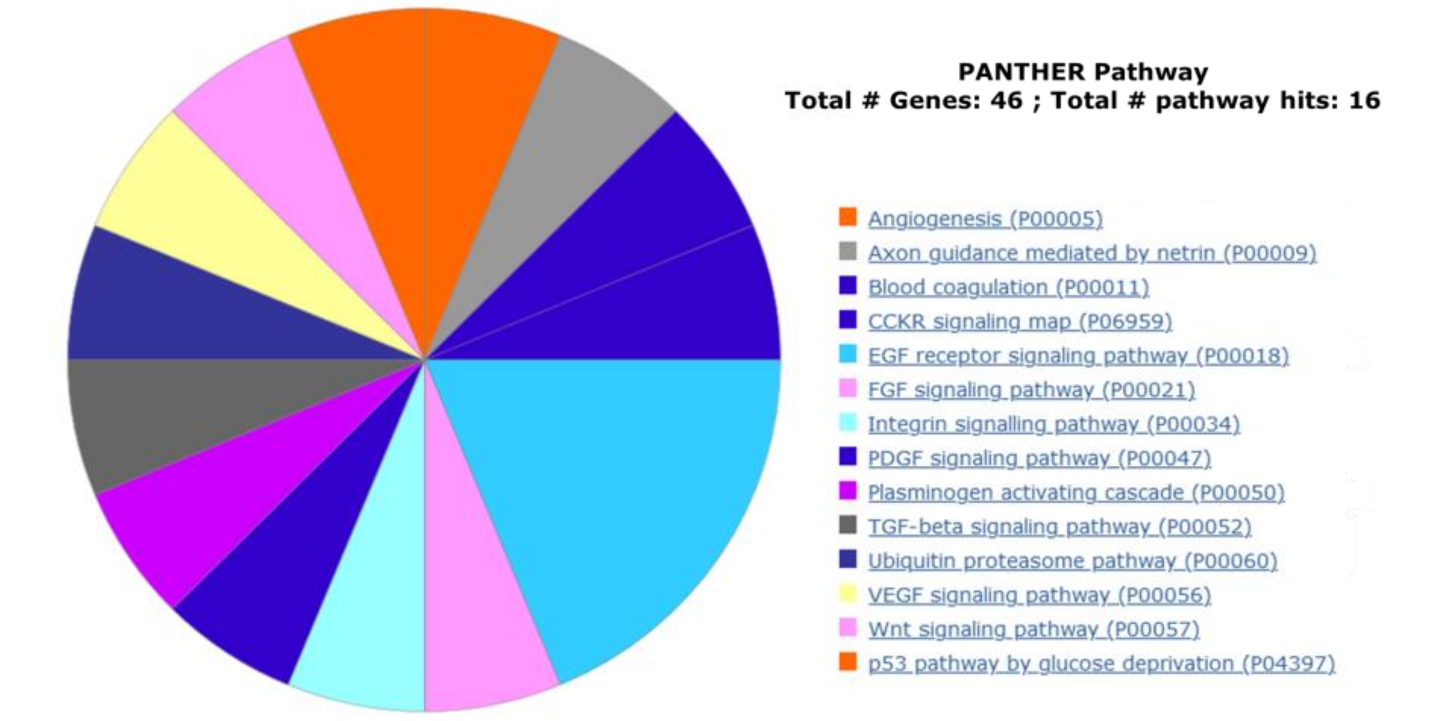

Supplementary Figure S2. Pie charts of GO-Slim analysis based on EBOV-miR-MAK-403 and EBOV-miR-MAY-251 mRNA targets. EBOV-miR-MAK-403 (**A**) and EBOV-miR-MAY-251 (**B**) mRNA targets with score >80 were subjected to the publicly available database PANTHER for gene function analysis. The "panther pathway" and "protein class" ontologies are represented here and complete the data set shown in **Figure 3**.
