# Supplementary material for: Ebola Virus Encodes Two microRNAs in Huh7-Infected Cells
Source: Int J Mol Sci. 2022 May 7;23(9):5228. doi: 10.3390/ijms23095228 (PMC9106010; doi:10.3390/ijms23095228)
Supplement: Supplementary file 1 [file ijms-23-05228-s001.zip › Suppl_File_S1_Alignement_miR-MAY-251_miR-MAK-403.pdf]

A) miR-MAY-251

Provisional ID	:	chrNC002549_251
Score total	:	862.5
Score for star read(s)	:	3.9
Score for read counts	:	878
Score for mfe	:	-16.6
Score for randfold	:	-2.2
Score for cons. seed	:	-0.6
Total read count	:	1734
Mature read count	:	1712
Loop read count	:	0
Star read count	:	22

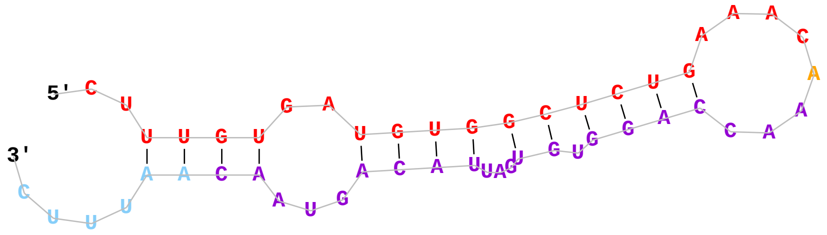

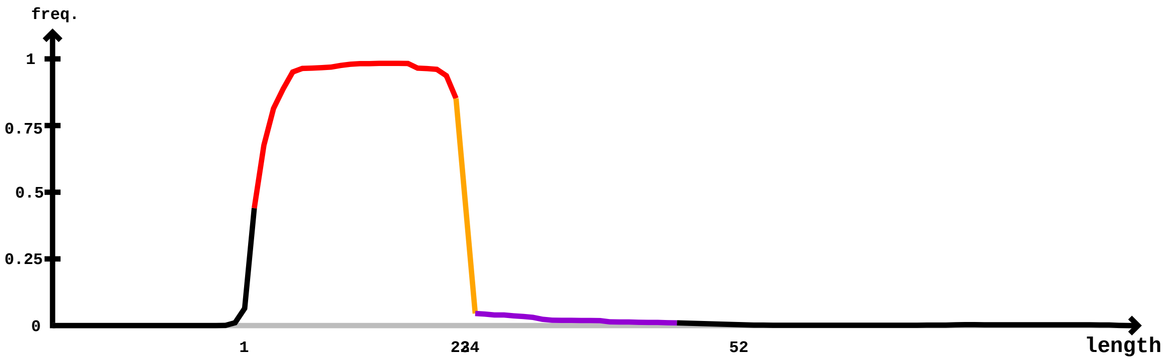

Mature **Star**

```
5'- gaccuagguuguucucuaucuuugugauguggcucugaaacaaaccaggugugauuacaguaacaauuucaauuuaaauuccgauauaaauuucaaugagaggaaaauuau  -3'   obs
    gaccuagguuguucucuaucuuugugauguggcucugaaacaaaccaggugugauuacaguaacaauuucaauuuaaauuccgauauaaauuucaaugagaggaaaauuau        exp
    ......((((.((((((....((((..((((((((((........)))).))...))))...))))...............((........))....)))))).))))..   reads    mm     sample
    .................aucuuugugauguggcucu.............................................................................   1        0      S23
    .................aucuuugugauguggcucugaaa.........................................................................   1        0      S23
    .................aucuuugugauguggcucugaaac........................................................................   4        0      S23
    .................aucuGugugauguggcucugaaac........................................................................   1        1      S23
    .................aucuuugugauguCgcucugaaacaa......................................................................   1        1      S23
    .................aucuuugugauguggcucugaaacaaa.....................................................................   1        0      S23
    ..................ucuuugugauguggcuc..............................................................................   1        0      S23
    ..................ucuuugugauguggcucugaaa.........................................................................   2        0      S23
    ..................Gcuuugugauguggcucugaaac........................................................................   1        1      S23
    ..................ucuuugugauguggcucugaaac........................................................................   36       0      S23
    ..................ucuuugugauguggcucugaaaG........................................................................   1        1      S23
    ..................ucuuugugauguggcucugaaacU.......................................................................   2        1      S23
    ..................ucuuugugauguggcucugaaacaa......................................................................   1        0      S23
    ..................ucuuugugauguggcucugaaacaaaccag.................................................................   1        0      S23
    ...................cuuugugauguggcucu.............................................................................   1        0      S23
    ...................cuuugugauguggcucugaa..........................................................................   6        0      S23
    ...................cuuugugauguggcucugaC..........................................................................   1        1      S23
    ...................cuuugugauguggcucugaaa.........................................................................   15       0      S23
    ...................cuuugugauguggcucugaCac........................................................................   1        1      S23
    ...................cuuugugauguggcucugaaaA........................................................................   2        1      S23
    ...................cuuugugauguggcuAugaaac........................................................................   1        1      S23
    ...................cuuugugaugAggcucugaaac........................................................................   1        1      S23
    ...................cuuugugaugugUcucugaaac........................................................................   1        1      S23
    ...................Uuuugugauguggcucugaaac........................................................................   1        1      S23
    ...................cuuugugaugugCcucugaaac........................................................................   1        1      S23
    ...................cuuugugaAguggcucugaaac........................................................................   1        1      S23
    ...................cuuugugauguggcucugaaac........................................................................   202      0      S23
    ...................cuuuguCauguggcucugaaac........................................................................   1        1      S23
    ...................Guuugugauguggcucugaaac........................................................................   5        1      S23
    ...................cuuugugauguggcucugaaaca.......................................................................   5        0      S23
    ...................cuuugugauguggAucugaaaca.......................................................................   1        1      S23
    ...................cuuugugauguggcucugaaacC.......................................................................   1        1      S23
    ...................cuuugugauguggcucugaaacaa......................................................................   3        0      S23
```

Mature **Star**

```
gaccuagguuguucucuaucuuugugauguggcucugaaacaaaccaggugugauuacaguaacaauuucaauuuaaauuccgauauaaauuucaaugagaggaaaauuau
...................cuuugugauguggcucugaaacaaacc.................................................................  2     0    S23
...................cuuugugauguggcucugaaacaaaccag...............................................................  1     0    S23
...................cuuugugauguggcucugaaacaaaccagg..............................................................  2     0    S23
....................uuugugauguggcucugaa........................................................................  3     0    S23
....................Guugugauguggcucugaaa.......................................................................  1     1    S23
....................uuugugauguggcucugaaa.......................................................................  13    0    S23
....................uuugugauCuggcucugaaac......................................................................  1     1    S23
....................uuugugauguggcucugaGac......................................................................  1     1    S23
....................uuugugauguggcucugaaac......................................................................  158   0    S23
....................uuugugauguggcucAgaaac......................................................................  1     1    S23
....................uuugugauguggcucugaaaA......................................................................  2     1    S23
....................Guugugauguggcucugaaac......................................................................  1     1    S23
....................uuugugauguggcucugaaacG.....................................................................  1     1    S23
....................uuugugauguggcucugaaaca.....................................................................  11    0    S23
....................uuugugauguggcucugaaacU.....................................................................  1     1    S23
....................uuugugauguggcucugaaaAa.....................................................................  1     1    S23
....................uuugugauguggcucugaaacaa....................................................................  1     0    S23
....................uuugugauguggcucugaaacaGa...................................................................  1     1    S23
....................uuugugauguggcucugaaacaaa...................................................................  2     0    S23
....................uuugugauguggcucugaaacaaaccag...............................................................  1     0    S23
....................uuugugauguggcucugaaacaaaccagg..............................................................  1     0    S23
.....................uugugauguggcucuga.........................................................................  2     0    S23
.....................uugugauguggcucugaa........................................................................  5     0    S23
.....................uugugauguggcucugaaa.......................................................................  6     0    S23
.....................uugugauguggcucugaaU.......................................................................  2     1    S23
.....................uugugauguggcucugaaaU......................................................................  1     1    S23
.....................Gugugauguggcucugaaac......................................................................  2     1    S23
.....................uugAgauguggcucugaaac......................................................................  1     1    S23
.....................uugugauguggcucugaaac......................................................................  66    0    S23
.....................Augugauguggcucugaaac......................................................................  1     1    S23
.....................uugugauguggcucugaaaca.....................................................................  1     0    S23
.....................uugugauguggcucugaaacU.....................................................................  3     1    S23
.....................uugugauguggcucugaaacaa....................................................................  1     0    S23
.....................uugugauguggcucugaaacaaacc.................................................................  1     0    S23
.....................uugugauguggcucugaaaAaaaccag...............................................................  1     1    S23
.....................uugugauguggcucugaaacaaaccagg..............................................................  1     0    S23
......................ugugauguggcucugaa........................................................................  3     0    S23
......................ugugauguggcucugaaa.......................................................................  11    0    S23
......................Ggugauguggcucugaaac......................................................................  1     1    S23
......................ugugauguggGucugaaac......................................................................  1     1    S23
......................ugugauguggcucugaaac......................................................................  41    0    S23
......................ugugauguggcucugaaaU......................................................................  1     1    S23
......................ugugauguggcucugaaacU.....................................................................  3     1    S23
......................ugugaugugCcucugaaaca.....................................................................  1     1    S23
.......................gugauguggcucugaaa.......................................................................  8     0    S23
.......................gugauguggcucugaaac......................................................................  33    0    S23
.......................gCgauguggcucugaaac......................................................................  1     1    S23
.......................gugauguggcucugaaaca.....................................................................  3     0    S23
.......................gugauguggcucugaaacU.....................................................................  1     1    S23
.......................gugauguggcucugaaacaaa...................................................................  1     0    S23
.......................gugauguggcucugaaacaaac..................................................................  1     0    S23
.......................gugauguggcucugaaacaaacU.................................................................  1     1    S23
........................ugauguggcucugaaac......................................................................  9     0    S23
........................ugauguggcucugaaaAa.....................................................................  2     1    S23
........................ugauguggcucugaaacaaacc.................................................................  1     0    S23
..........................auguggcucugaaacaaaccaggu.............................................................  1     0    S23
..........................auguggcucugaaacaaaccaggugugau........................................................  1     0    S23
...........................uguggcucugaaacaaaccagg..............................................................  1     0    S23
...........................uguggcucugaaacaaaccaggugugauu.......................................................  2     0    S23
............................guggcucugaaacaaaccaggugugauu.......................................................  3     0    S23
.............................uggcucugaaacaaaccaggugugauua......................................................  1     0    S23
................................cucugaaacaaaccaggugugauuaca....................................................  1     0    S23
.........................................aaaccaggugugauuacaguaaca..............................................  1     0    S23
..........................................aaccaggugugauuacaguaac...............................................  2     0    S23
...........................................accaggugugauuacaguaac...............................................  1     0    S23
............................................ccaggugugauuacagua.................................................  1     0    S23
.............................................caggugugauuacaguaac...............................................  1     0    S23
....................................................gauuacaguaacaauuucaauu.....................................  1     0    S23
.............................................................................................caaugagaggaaaauua.  1     0    S23

................uaucuuugugauguggcucugaC........................................................................  1     1    S15
```

Mature Star

```
gaccuagguuguucucuaucuuugugauguggcucugaaacaaaccaggugugauuacaguaacaauuucaauuuaaauuccgauauaaauuucaaugagaggaaaauuau
.................aucuuugugauguggcucugaaaU......................................................................  2    1  S15
.................aucuuugugauguggcucugaaac......................................................................  6    0  S15
.................aucuuugugauguggcucugaaaca.....................................................................  1    0  S15
..................ucuuugugauguggcucC...........................................................................  1    1  S15
..................ucuuugugauguggcucu...........................................................................  3    0  S15
..................Gcuuugugauguggcucugaaac......................................................................  1    1  S15
..................ucuuuguUauguggcucugaaac......................................................................  2    1  S15
..................ucuuugugauguggcucugaaaU......................................................................  1    1  S15
..................ucuuugugauguggcucugGaac......................................................................  1    1  S15
..................ucuuugugauguggcucugaaac......................................................................  36   0  S15
..................ucuuugugauguggcucugaaaA......................................................................  1    1  S15
..................ucuuugugauguggcucugaaaca.....................................................................  3    0  S15
..................ucuuugugauguggcucugCaaca.....................................................................  1    1  S15
...................cuuugugauguggcuAu...........................................................................  1    1  S15
...................cuuugugauguggcucu...........................................................................  15   0  S15
...................cuCugugauguggcucu...........................................................................  1    1  S15
...................cuuugugauguggcucA...........................................................................  4    1  S15
...................cuuugCgauguggcucu...........................................................................  1    1  S15
...................cuuugugauguggcuUu...........................................................................  1    1  S15
...................Guuugugauguggcucu...........................................................................  1    1  S15
...................cuuugugauguggcucC...........................................................................  2    1  S15
...................cuuugugauguggcucAg..........................................................................  1    1  S15
...................cuuugugauguggcucugaa........................................................................  5    0  S15
...................cuCugugauguggcucugaaa.......................................................................  1    1  S15
...................cuuugugauguggcucugaaG.......................................................................  1    1  S15
...................Uuuugugauguggcucugaaa.......................................................................  1    1  S15
...................cuuugugauguggcucugaaa.......................................................................  19   0  S15
...................cuuugugauguggcucugaUac......................................................................  1    1  S15
...................cuuugugauguggcucugaCac......................................................................  1    1  S15
...................cuuugugauUuggcucugaaac......................................................................  1    1  S15
...................cuuuguUauguggcucugaaac......................................................................  1    1  S15
...................cuuugugauguggcucugaaaU......................................................................  16   1  S15
...................cuuugugauguggcucuCaaac......................................................................  1    1  S15
...................cuuugugUuguggcucugaaac......................................................................  1    1  S15
...................cuuugugaCguggcucugaaac......................................................................  1    1  S15
...................cuuugugauAuggcucugaaac......................................................................  1    1  S15
...................cuuCgugauguggcucugaaac......................................................................  1    1  S15
...................Auuugugauguggcucugaaac......................................................................  2    1  S15
...................cuuugugauguggcuAugaaac......................................................................  1    1  S15
...................cuuugugauguggcucCgaaac......................................................................  2    1  S15
...................cuuugugauguggcucugCaac......................................................................  2    1  S15
...................Guuugugauguggcucugaaac......................................................................  1    1  S15
...................cuuugugauguggcucugUaac......................................................................  1    1  S15
...................cuuugugaugugUcucugaaac......................................................................  2    1  S15
...................cuuugugauguAgcucugaaac......................................................................  1    1  S15
...................cuuugugauguggAucugaaac......................................................................  1    1  S15
...................cuuugugauguggcucugaGac......................................................................  2    1  S15
...................cuuugugGuguggcucugaaac......................................................................  2    1  S15
...................cuuugugauguggcucugaaaA......................................................................  5    1  S15
...................cuuugugaugGggcucugaaac......................................................................  2    1  S15
...................cuuugugaugCggcucugaaac......................................................................  3    1  S15
...................cuuugugauguggUucugaaac......................................................................  2    1  S15
...................cuuugCgauguggcucugaaac......................................................................  1    1  S15
...................cuGugugauguggcucugaaac......................................................................  1    1  S15
...................cuuugugauguggcucugaaac......................................................................  290  0  S15
...................cuuugugauguggcucugaGaca.....................................................................  1    1  S15
...................cuuugugauguggcucugaaaca.....................................................................  9    0  S15
...................cuuugugauguggcucugaaacU.....................................................................  1    1  S15
...................cuuugugauguggcucugaaacaaa...................................................................  1    0  S15
....................uuugugauguggcucug..........................................................................  2    0  S15
....................uuugugauguggcucuga.........................................................................  3    0  S15
....................uuugugauguggcucugaa........................................................................  13   0  S15
....................uuuguUauguggcucugaaa.......................................................................  1    1  S15
....................uuugugauguggcucugaaa.......................................................................  20   0  S15
....................uuugugauguggcucuUaaac......................................................................  3    1  S15
....................uuugugauguggUucugaaac......................................................................  1    1  S15
....................uuCgugauguggcucugaaac......................................................................  2    1  S15
....................uuugugauguggcucugaaGc......................................................................  1    1  S15
....................Guugugauguggcucugaaac......................................................................  2    1  S15
....................uuugugauguggcucugaaCc......................................................................  1    1  S15
```

Mature **Star**

gaccuagguuguucucuaucuuugugauguggcucugaaacaaaccaggugugauuacaguaacaauuucaauuuaaauuccgauauaaauuucaaugagaggaaaauuau			
....................uuuCugauguggcucugaaac..	1	1	S15
....................uuugugaugCggcucugaaac..	1	1	S15
....................uuugugauguggcucugaaaA..	2	1	S15
....................uuugugauguggcucugGaac..	1	1	S15
....................uuugugauguggcucugCaac..	1	1	S15
....................uuugugaGguggcucugaaac..	1	1	S15
....................uuugugauguggcucugaaac..	138	0	S15
....................uuugugauguggcCcugaaac..	1	1	S15
....................uuuguUauguggcucugaaac..	3	1	S15
....................uuugugauguggcucugaaaU..	9	1	S15
....................uuugugauguggcucugaGac..	1	1	S15
....................uuugugaugugCcucugaaac..	1	1	S15
....................uAugugauguggcucugaaac..	1	1	S15
....................uuugugauguggcucGgaaac..	1	1	S15
....................uuugugauguggcucugaaaca...	4	0	S15
....................uuugugauguggcucugaaaAa...	2	1	S15
....................uuugugauguggcucugaaacG...	1	1	S15
....................uuugugauguggcCcugaaacaa..	1	1	S15
....................uuuguUauguggcucugaaacaaa...	1	1	S15
.....................uugugauguggcucugaa..	5	0	S15
.....................Augugauguggcucugaaa...	1	1	S15
.....................uugugGuguggcucugaaa...	1	1	S15
.....................uuCugauguggcucugaaa...	1	1	S15
.....................uugugauguggcucCgaaa...	1	1	S15
.....................uugugauguggcucugaaa...	23	0	S15
.....................uugugauguggcucugaaGc..	2	1	S15
.....................uuguAauguggcucugaaac..	1	1	S15
.....................uugugauguggcucugaaaU..	6	1	S15
.....................uugugauguggcucugaaCc..	1	1	S15
.....................uugugauguggcucugGaac..	1	1	S15
.....................uugugauUuggcucugaaac..	1	1	S15
.....................uugugauguggcucuUaaac..	1	1	S15
.....................uugugauguggcucugaaUc..	1	1	S15
.....................uugugauguggcucugaaaA..	10	1	S15
.....................uugugauguggAucugaaac..	1	1	S15
.....................Gugugauguggcucugaaac..	2	1	S15
.....................uugugauguggcucugaaac..	83	0	S15
.....................Augugauguggcucugaaac..	2	1	S15
.....................uugugauguggcucugaaacC...	1	1	S15
.....................uugugauguggcucugaaaAa...	1	1	S15
.....................uugugauguggcucugaaaca...	4	0	S15
.....................uugugauguggcucugaaacaaa...	2	0	S15
......................ugugauguggcucugaa..	1	0	S15
......................Ggugauguggcucugaaa...	1	1	S15
......................ugugauguggcucugaaa...	8	0	S15
......................ugugauguggcucugaGac..	1	1	S15
......................uAugauguggcucugaaac..	1	1	S15
......................ugugauguggcucugaaaA..	4	1	S15
......................ugugauguggcucugaaac..	39	0	S15
......................ugugauguggcucugaCac..	1	1	S15
......................ugugauguggcucugaaaU..	5	1	S15
......................ugugauguggcucugaaaca...	4	0	S15
......................Agugauguggcucugaaaca...	1	1	S15
......................ugugauguggcucugaaaAa...	1	1	S15
......................ugugauguggcucugaaacaa..	1	0	S15
......................ugugauguggcucugaaacaaaccaggG...	1	1	S15
.......................Uugauguggcucugaaa...	1	1	S15
.......................gugauguggcucugaaa...	9	0	S15
.......................gugauguUgcucugaaac..	1	1	S15
.......................gugUuguggcucugaaac..	1	1	S15
.......................gugauguggcucugaaac..	41	0	S15
.......................gugauguggcucugaGac..	1	1	S15
.......................gugauguggcucugaaaA..	2	1	S15
.......................gugauguggcucugaaaU..	5	1	S15
.......................gugauguggcucugaaacGa..	1	1	S15
.......................gugauguggcucugaaacaaaccaggugug..	1	0	S15
........................ugauguggcucugaaaU..	1	1	S15
........................ugauguggcucugaaac..	5	0	S15
........................ugauguggcucugaaCc..	1	1	S15
........................ugauguggcucugCaac..	2	1	S15

Mature **Star**

```
gaccuagguuguucucuaucuuugugauguggcucugaaacaaaccaggugugauuacaguaacaauuucaauuuaaauuccgauauaaauuucaaugagaggaaaauuau
........................ugauguggcucugaaaca.....................................................................  2  0  S15
.........................gauguggcucugaaacaaaccaggC.............................................................  1  1  S15
..........................auguggcucugaaacaaaccaggugu...........................................................  1  0  S15
............................guggcucugaaacaaaccaggugugauu.......................................................  1  0  S15
..............................ggcucugaaacaaaccaggugugGuu.......................................................  1  1  S15
................................cucugaaacaaaccaggugugauu.......................................................  1  0  S15
..........................................aaccaggugugauuacag...................................................  1  0  S15
..........................................aaccaggugugauuacaguaac...............................................  1  0  S15
...........................................accaggugugauuacaguaac...............................................  2  0  S15
............................................ccaggugugauuacaguaaU...............................................  1  1  S15
..........................................................................uaaauuccgauauaaauuuca................  1  0  S15
.........................................................................................auuucaaugagaggaaaauu..  1  0  S15
...........................................................................................uucaaugagaggaaaa....  1  0  S15

.......................gugauAuggcucugaaac......................................................................  1  1  S13
.......................gugauAuggcucugaaaca.....................................................................  1  1  S13
........................ugauAuggcucugaaacaaaccag...............................................................  1  1  S13
.........................gauAuggcucugaaacaaacc.................................................................  1  1  S13
.............................uggcucugaaacaaaccagg..............................................................  1  0  S13
.............................uggcucugaaacaaaccaggu.............................................................  1  0  S13
.............................uggcucugaaacaaaccaggug............................................................  1  0  S13
..............................ggcucugaaacaaaccag...............................................................  1  0  S13
..............................ggcucugaaacaaaccagg..............................................................  1  0  S13
....................................gaaacaaaccagguAugauuaca....................................................  1  1  S13
.........................................aaaccagguAugauuacaguaac...............................................  1  1  S13
.........................................aaaccagguAugauuacaguaaca..............................................  1  1  S13
..........................................aaccagguAugauuacagua.................................................  1  1  S13
..........................................aaccagguAugauuacaguaac...............................................  2  1  S13
...........................................accagguAugauuacaguaa................................................  1  1  S13
...........................................accagguAugauuacaguaac...............................................  2  1  S13
............................................ccagguAugauuacaguaac...............................................  1  1  S13
..................................................Augauuacaguaacaauuucaau......................................  1  1  S13
.......................................................................auuuaaauuccgauauaaa.....................  1  0  S13
............................................................................................ucaaugagaggaaaauu..  2  0  S13

...................cuuugugauguggcucugaaac......................................................................  1  0  S21
...........................uAuggcucugaaacaaaccagg..............................................................  1  1  S21
............................Auggcucugaaacaaaccagg..............................................................  4  1  S21
............................Auggcucugaaacaaaccaggu.............................................................  3  1  S21
.............................uggcucugaaacaaacca................................................................  4  0  S21
..............................ggcucugaaacaaaccagg..............................................................  1  0  S21

..................ucuuugugauguggcucugaaac......................................................................  1  0  S14
...................cuuugugauguggcucugaaa.......................................................................  4  0  S14
```

B) miR-MAK-403

Provisional ID	:	chrKJ660347_403
Score total	:	249.1
Score for star read(s)	:	3.9
Score for read counts	:	255
Score for mfe	:	-6.9
Score for randfold	:	-2.2
Score for cons. seed	:	-0.6
Total read count	:	512
Mature read count	:	397
Loop read count	:	112
Star read count	:	3

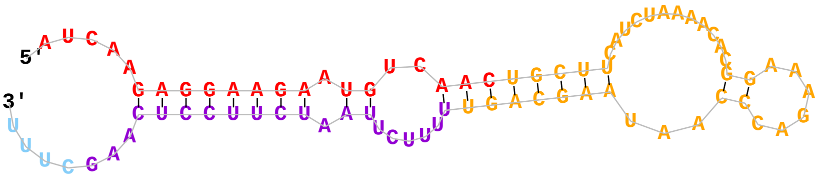

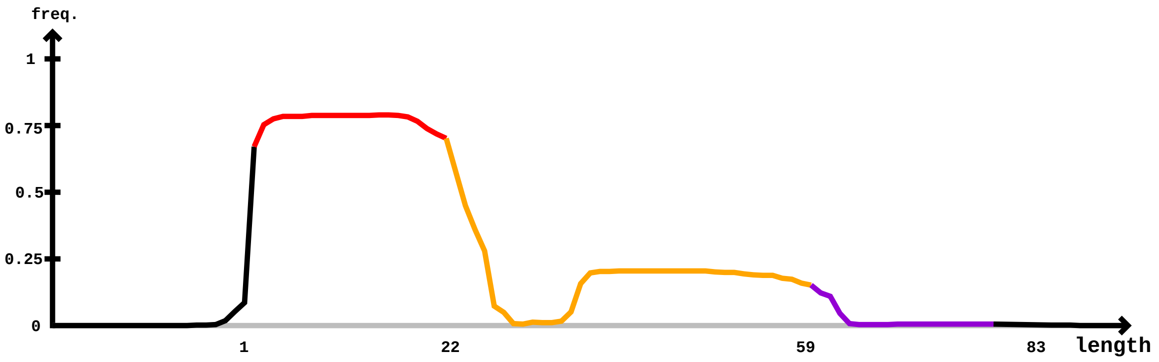


```
                         Mature                                                         Star
5'- uguuguguagccauuuaauaucaagaggaagaaugucaacugcuucaucuaaaacacggaaagacccaauaagcaguuuuucuuaaucuucccucaagcuuuuuucuuaac   -3'   obs
    uguuguguagccauuuaauaucaagaggaagaaugucaacugcuucaucuaaaacacggaaagacccaauaagcaguuuuucuuaaucuucccucaagcuuuuuucuuaac         exp
    ........................((((((((.((..((((((((............((......))...)))))))).....)).))))))))).................    reads   mm   sample
    .................auaucaagaggaagaauUuca.........................................................................    1       1    S23
    .................auaucaagaggaagaauUucaacug.....................................................................    1       1    S23
    ...................aucaagaggaagaauUu...........................................................................    2       1    S23
    ...................aucaagaggaagaauUucaacugcu...................................................................    1       1    S23
    ...................aucaagaggaagaauUucaacugcuu..................................................................    3       1    S23
    ....................ucaagaggaagaauUucaacu......................................................................    1       1    S23
    .....................caagaggaagaauUucaacugcu...................................................................    1       1    S23
    ...............................................ucuaaaacacggaaagaccca...........................................    1       0    S23
    ....................................................aacacggaaagacccaauaagc.....................................    1       0    S23
    ....................................................aacacggaaagacccaauaagcaguuuu...............................    2       0    S23
    ....................................................aacacggaaagacccaauaagcaguuuuu..............................    2       0    S23
    .....................................................acacggaaagacccaGuaagcagu..................................    1       1    S23
    .....................................................acacggaaagacccaauaagcaguu.................................    3       0    S23
    .....................................................Gcacggaaagacccaauaagcaguuu................................    1       1    S23
    .....................................................acacggaaagCcccaauaagcaguuu................................    1       1    S23
    .....................................................acacggaaagacccaauaagcaguuuu...............................    6       0    S23
    .....................................................acacggaaagacccaauaagcaguuuuu..............................    5       0    S23
    ......................................................cacggaaagacccaauaagcaguu.................................    1       0    S23
    ......................................................cacggaaagacccaauaagcaguuuu...............................    2       0    S23
    ......................................................cacggaaagacccaauaagcaguuuuA..............................    1       1    S23
    ......................................................cacggaaagacccaauaagcaguuuuuA.............................    2       1    S23
    .......................................................acggaaagacccaauaagcaguuuu...............................    2       0    S23
    .......................................................acggaaagacccaauaagcaguuuuu..............................    1       0    S23
    .........................................................ggaaagacccaauaagcaguu.................................    1       0    S23

    .................auaucaagaggaagaauU............................................................................    1       1    S15
    ...................aucaagaggaagaauUu...........................................................................    3       1    S15
    ....................ucaagaggaagaauUucaacugcu...................................................................    3       1    S15
    ................................auUucaacugcuucauc..............................................................    1       1    S15
    ....................................................aacacggaaagacccaaua........................................    1       0    S15
    ....................................................aacacggaaagacccaauaagc.....................................    1       0    S15
    ....................................................aacacggaaagacccaauaagcag...................................    1       0    S15
    ....................................................aacacggaaagGcccaauaagcaguu.................................    1       1    S15
```

Mature **Star**

uguuguguagccauuuaauaucaagaggaagaaugucaacugcuucaucuaaaacacggaaagacccaauaagcaguuuuucuuaaucuuccucaagcuuuuuucuuaac			
....aacacggaaagacccaauaagcaguuuu..............................	1	0	S15
....acacggaaagacccaauaagca...................................	1	0	S15
....acacggaaagacccaauaagcaguu................................	3	0	S15
....acacggaaagacccaauaagcaguA................................	1	1	S15
....acacggaaagacccaauaagcaguuuu..............................	2	0	S15
....acacggaaagacccaauaagcaguuuuC.............................	1	1	S15
....acacggaaagacccaauaagcaguuuuu.............................	2	0	S15
....cacggaaagacccaaua.......................................	1	0	S15
....cacggaaagacccaauaagcaguA................................	1	1	S15
....cacggaaagacccaauaagcaguu................................	1	0	S15
....cacggaaagCccaauaagcaguuuuu..............................	1	1	S15
....ucuuccucaGgcuuuuuuc.....	1	1	S15
.............uuuaauaucaagaggaagaauguA...	1	1	S13
...............uaauaucaagaggaagaaug...	1	0	S13
................aauaucaagaggaagaau..	1	0	S13
................aauaucaagaggaagaaugucaac..	4	0	S13
................aauaucaagaggaagaaugucaacugcuu..	1	0	S13
.................auaucaagaggaagaaug...	1	0	S13
.................auaucaagaggaagaauguA...	1	1	S13
.................auaucaagaggaagaauguc...	1	0	S13
.................auaucaagaggaagaaugGcaac..	1	1	S13
.................auaucaagaggaagaaugucaac..	1	0	S13
.................auaucaagaggaagaaGgucaac..	1	1	S13
.................auaucaagaggaagaaugucaacug..	1	0	S13
.................auaucaagaggaagaaugucaacugcuu...	1	0	S13
..................uaucaagaggaagaauguc...	1	0	S13
..................uaucaagaggaagaaugucaa...	1	0	S13
..................uaucaagaggaagaaugucaac..	4	0	S13
..................uaucaagaggaagaaugucaacu...	2	0	S13
..................uaucaagaggaagaaugucaacug..	2	0	S13
..................uaucaagaggaagaaugucaacugA...	1	1	S13
..................uaucaagaggaagaaugucaacugc...	1	0	S13
..................uaucaagaggaagaaugucaacugcu..	3	0	S13
..................uaucaagaggaagaaugucaacugcuuc..	1	0	S13
...................aucaagaggaagaaugC..	1	1	S13
...................aucaagaggaagaaugu..	3	0	S13
...................aucaagaggaagaauguc...	8	0	S13
...................aucaagaggaagaaugucU..	1	1	S13
...................aucaagaggaagaauguca..	4	0	S13
...................aucaagaggaagaaugucaa...	4	0	S13
...................aGcaagaggaagaaugucaac..	1	1	S13
...................aucaagaggaagaaugucaaA..	1	1	S13
...................aucaagaggaagaaugucaac..	50	0	S13
...................aucaagaggaGgaaugucaac..	1	1	S13
...................aCcaagaggaagaaugucaac..	1	1	S13
...................aucaagaggaagaaugucGac..	1	1	S13
...................aucaagaggaagaaugucaaU..	6	1	S13
...................aAcaagaggaagaaugucaac..	1	1	S13
...................aucaagaggaagaaugucaacu...	19	0	S13
...................aucaagaggaagaaugucaacA...	2	1	S13
...................aucaagaggaagaauguAaacu...	2	1	S13
...................aucaagaggaagaaugucaCcu...	1	1	S13
...................aucaagaggaagaaugucaacG...	1	1	S13
...................aucaagaggaagaaugCcaacu...	1	1	S13
...................aucaagaggaagaaugucaacC...	2	1	S13
...................aucaagaggaagaaugucaacug..	21	0	S13
...................aucaagaggaagaaugucaacuA..	3	1	S13
...................aucaagaggaagaaugucaacuC..	1	1	S13
...................aucaagaggaagaaugucaacugU...	5	1	S13
...................aucaagaggaagaaugucaacugc...	14	0	S13
...................aucaagaggaagaaugucaacugA...	4	1	S13
...................aucaagaggaagaauguAaacugc...	1	1	S13
...................aucaagaggaagaaugucaCcugcu..	1	1	S13
...................aucaagaggaagaaugucaacugcC..	11	1	S13
...................aucaagaggaagaaugucaacuAcu..	1	1	S13
...................aucaagaggaagaaCgucaacugcu..	1	1	S13
...................aucaagaggaagaaugucaacugUu..	1	1	S13
...................aucaagaggaagaaugucaacugcu..	49	0	S13
...................aucaagaggaagaaugucUacugcu..	1	1	S13

```
                   Mature                                                    Star
uguuguguagccauuuaauaucaagaggaagaaugucaacugcuucaucuaaaacacggaaagacccaauaagcaguuuucuuaaucuuccucaagcuuuuuucuuaac
...................aucaagaggaagaaugucaacugcG.................................................................   5    1    S13
...................aucaagaggaagaaugucaacugcA.................................................................   6    1    S13
...................aCcaagaggaagaaugucaacugcu.................................................................   1    1    S13
...................aucaagaggaagaaugucaacugcuu................................................................   3    0    S13
...................aucaagaggaagaaugucaacugcuuc...............................................................   3    0    S13
...................aCcaagaggaagaaugucaacugcuuc...............................................................   1    1    S13
....................ucaagaggaagaauguca.......................................................................   1    0    S13
....................ucaagaggaagaaugucaac.....................................................................   4    0    S13
....................ucaagaggaagaaugucaacu....................................................................   1    0    S13
....................ucaagaggaagaaugucaacuU...................................................................   1    1    S13
....................ucaagaggaagaaugucaacuA...................................................................   1    1    S13
....................ucaagaggaagaaugucaacug...................................................................   4    0    S13
....................ucaagaggaagaaugucaacugA..................................................................   1    1    S13
....................ucaagaggaagaaugucaacugc..................................................................   1    0    S13
....................ucaagaggaagaaugucaacugcA.................................................................   1    1    S13
....................ucaagaggaagaaugucaacugcC.................................................................   2    1    S13
....................ucaagaggaagaaugucaacugcu.................................................................   10   0    S13
.....................caagaggaagaaugucaac.....................................................................   2    0    S13
.....................caagaggaagaaugucaacuA...................................................................   2    1    S13
.....................caagaggaagaaugucaacugcu.................................................................   1    0    S13
......................aagaggaagaaugucaac.....................................................................   1    0    S13
......................aagaggaagaaugucaacug...................................................................   1    0    S13
......................aagaggaagaaugucaacugc..................................................................   1    0    S13
.........................aggaagaaugucaacugcuC................................................................   1    1    S13
.........................aggaagaaugucaacugcuu................................................................   1    0    S13
..............................................aucuaaaacacggaaagaccc..........................................   1    0    S13
................................................cuaaaacacggaaagaccA..........................................   1    1    S13
...................................................aaacacggaaagacccaauaagca..................................   1    0    S13
....................................................aacacggaaagacccaauaagc...................................   1    0    S13
....................................................aacacggaaagacccaauaagcagu................................   1    0    S13
....................................................aacacggaaagacccaauaagcaguu...............................   1    0    S13
....................................................aacacggaaagacccaauaagcaguG...............................   1    1    S13
....................................................aacacggaaagacccaauaagcaguuuu.............................   3    0    S13
....................................................aacacggaaagacccaauaagcaguuuCu............................   1    1    S13
.....................................................acacggaaagacccaauaa.....................................   1    0    S13
.....................................................acacggaaagacccaauaagc...................................   1    0    S13
.....................................................acacggaaagaccAaauaagcagu................................   1    1    S13
.....................................................acacggaaagacccaauaagcaguu...............................   2    0    S13
.....................................................acacggaaagacccaauaagcaguuu..............................   2    0    S13
.....................................................acacggaaagacccaauaagcaguuA..............................   1    1    S13
.....................................................acacggaaagacccaauaagcaguuuu.............................   11   0    S13
.....................................................acacggaaagacccaauaagcaguuuC.............................   1    1    S13
.....................................................acacgUaaagacccaauaagcaguuuuu............................   1    1    S13
.....................................................acacggaaagacccaauaagcaguuuuu............................   2    0    S13
......................................................cacggaaagacAcaauaagcagu................................   1    1    S13
......................................................cacggaaagGcccaauaagcagu................................   1    1    S13
......................................................cacggaaagacccaauaagcagu................................   1    0    S13
......................................................cacggaaagacccaauaagcaguuu..............................   2    0    S13

................aauaucaGgaggaagaaugucaacugcuuc...............................................................   1    1    S21
................aauaucaagaggaagaaugucaacugcuuc...............................................................   1    0    S21
.................auaucaagaggaagaauguc........................................................................   1    0    S21
.................auaucaagaggaagaaugucaacug...................................................................   4    0    S21
.................auaucaagaggaagaaugucaacugcuu................................................................   2    0    S21
.................auaucaagaggaagaaugucaacugcuuc...............................................................   1    0    S21
..................uaucaagaggaagaaugucaac.....................................................................   2    0    S21
...................aucaagaggaagaauguc........................................................................   2    0    S21
...................aucaagaggaagaaugucG.......................................................................   4    1    S21
...................aucaaUaggaagaaugucaac.....................................................................   1    1    S21
...................aucaagaggaagaaugucaac.....................................................................   15   0    S21
...................aucaagaggaagaaugCcaac.....................................................................   1    1    S21
...................aucaagaggaagaaugucaacug...................................................................   7    0    S21
...................aucaagaggaagaaugucaacugc..................................................................   6    0    S21
...................aucaagaggaagaaugucaacugA..................................................................   3    1    S21
...................aucaagaggaagaaugucaacugcu.................................................................   7    0    S21
...................aucaagaggaUgaaugucaacugcu.................................................................   1    1    S21
...................aucaagaggaagaaugucaacugcA.................................................................   1    1    S21
...................aucaaUaggaagaaugucaacugcuu................................................................   1    1    S21
...................aucaagaggaagaaugucaacugcuuc...............................................................   15   0    S21
...................aucaagaggaGgaaugucaacugcuuc...............................................................   1    1    S21
```

```
                   Mature                                                         Star
uguuguguagccauuuaauaucaagaggaagaaugucaacugcuucaucuaaaacacggaaagacccaauaagcaguuuuucuuaaucuuccucaagcuuuuuucuuaac
...................aucaagaggaagaaugucaacugcuuca...............................................................   2   0   S21
....................ucaagaggaagaaugucaa.......................................................................   4   0   S21
....................ucaagaggaagaaugucaac......................................................................   4   0   S21
....................ucaagaggaagaaugucaacugA...................................................................   2   1   S21
....................ucaagaggaagaaugucaacugcu..................................................................   4   0   S21
.....................caagaggaagaaugucaac......................................................................   2   0   S21
.....................caagaggaagaaugucaacugU...................................................................   1   1   S21
.....................caagaggaagaaugucaacugA...................................................................   3   1   S21
......................aagaggaagaaugucaacu.....................................................................   1   0   S21
......................aagaggaagaaugucaacugcu..................................................................   1   0   S21
................................................cuaaaacacggaaagacccaaA........................................   3   1   S21
...................................................aaacacggaaagacccaauaagcaguuuuu.............................   2   0   S21
....................................................aacacggaaagacccaauaagcaguuuuu.............................   1   0   S21
.....................................................Ccacggaaagacccaauaagc....................................   2   1   S21
.....................................................acCcggaaagacccaauaagcaguuuu..............................   1   1   S21
.....................................................acacggaaagacccaauaagcaguuuu..............................   4   0   S21
.....................................................acacggaaagacccaauaagcaguuuuu.............................   1   0   S21
......................................................cacggaaagacccaauaagUag..................................   1   1   S21
......................................................cacggaaagacccaauaagcag..................................   5   0   S21
......................................................cCcggaaagacccaauaagcag..................................   1   1   S21
.............................................................................uuuucuuaaucuucAucaag.............   2   1   S21
```
