# Supplementary material for: Ebola Virus Encodes Two microRNAs in Huh7-Infected Cells
Source: Int J Mol Sci. 2022 May 7;23(9):5228. doi: 10.3390/ijms23095228 (PMC9106010; doi:10.3390/ijms23095228)
Supplement: Supplementary file 1 [file ijms-23-05228-s001.zip › Suppl_File_S2_list_of_miRNAs_Targets_miRDB.pdf]

A) miR-MAY-251

There are 881 predicted targets for the submitted 22 nt long miRNA: cuuugugauguggcucugaaac (miR-MAY-251)

Return to Custom Prediction

Target Detail	Target Rank	Target Score	miRNA Name	Gene Symbol	Gene Description
Details	1	99	submission	SGIP1	SH3 domain GRB2 like endophilin interacting protein 1
Details	2	99	submission	MBNL1	muscleblind like splicing regulator 1
Details	3	98	submission	DUSP16	dual specificity phosphatase 16
Details	4	98	submission	ASXL3	ASXL transcriptional regulator 3
Details	5	98	submission	NAMPT	nicotinamide phosphoribosyltransferase
Details	6	98	submission	FGD1	FYVE, RhoGEF and PH domain containing 1
Details	7	98	submission	PUM2	pumilio RNA binding family member 2
Details	8	98	submission	ADGRL2	adhesion G protein-coupled receptor L2
Details	9	98	submission	SCN2A	sodium voltage-gated channel alpha subunit 2
Details	10	97	submission	NOTCH2	notch 2
Details	11	97	submission	WDR20	WD repeat domain 20
Details	12	97	submission	WWC2	WW and C2 domain containing 2
Details	13	97	submission	PDXDC1	pyridoxal dependent decarboxylase domain containing 1
Details	14	97	submission	SLF2	SMC5-SMC6 complex localization factor 2
Details	15	96	submission	UNC5D	unc-5 netrin receptor D
Details	16	96	submission	CNTNAP2	contactin associated protein like 2
Details	17	96	submission	PLCB4	phospholipase C beta 4
Details	18	96	submission	TCTEX1D1	Tctex1 domain containing 1
Details	19	96	submission	ZC3H14	zinc finger CCCH-type containing 14
Details	20	96	submission	CAMTA1	calmodulin binding transcription activator 1
Details	21	96	submission	B3GNT9	UDP-GlcNAc:betaGal beta-1,3-N-acetylglucosaminyltransferase 9
Details	22	95	submission	DUSP19	dual specificity phosphatase 19
Details	23	95	submission	HMG20A	high mobility group 20A
Details	24	95	submission	TIA1	TIA1 cytotoxic granule associated RNA binding protein
Details	25	95	submission	THAP9	THAP domain containing 9
Details	26	95	submission	FZD3	frizzled class receptor 3

Details	27	95	submission	MAB21L2	mab-21 like 2
Details	28	95	submission	CLIP1	CAP-Gly domain containing linker protein 1
Details	29	95	submission	WBP4	WW domain binding protein 4
Details	30	95	submission	COL4A3	collagen type IV alpha 3 chain
Details	31	94	submission	NUSAP1	nucleolar and spindle associated protein 1
Details	32	94	submission	EEA1	early endosome antigen 1
Details	33	94	submission	RBM20	RNA binding motif protein 20
Details	34	94	submission	FCRL1	Fc receptor like 1
Details	35	94	submission	EPC1	enhancer of polycomb homolog 1
Details	36	94	submission	STEAP2	STEAP2 metalloreductase
Details	37	94	submission	ZBTB20	zinc finger and BTB domain containing 20
Details	38	93	submission	PTBP3	polypyrimidine tract binding protein 3
Details	39	93	submission	TSPOAP1	TSPO associated protein 1
Details	40	93	submission	OSBPL8	oxysterol binding protein like 8
Details	41	93	submission	TRDMT1	tRNA aspartic acid methyltransferase 1
Details	42	93	submission	FGF12	fibroblast growth factor 12
Details	43	93	submission	RBMS2	RNA binding motif single stranded interacting protein 2
Details	44	93	submission	PGAP1	post-GPI attachment to proteins 1
Details	45	93	submission	MTMR14	myotubularin related protein 14
Details	46	92	submission	ZBTB37	zinc finger and BTB domain containing 37
Details	47	92	submission	WDR44	WD repeat domain 44
Details	48	92	submission	SLC24A2	solute carrier family 24 member 2
Details	49	92	submission	MBNL3	muscleblind like splicing regulator 3
Details	50	92	submission	ADAM23	ADAM metallopeptidase domain 23
Details	51	92	submission	DGKH	diacylglycerol kinase eta
Details	52	92	submission	STT3B	STT3B, catalytic subunit of the oligosaccharyltransferase complex
Details	53	92	submission	E2F5	E2F transcription factor 5
Details	54	92	submission	SIX4	SIX homeobox 4
Details	55	92	submission	S100PBP	S100P binding protein
Details	56	91	submission	ADAMTSL1	ADAMTS like 1
Details	57	91	submission	MAP2K7	mitogen-activated protein kinase kinase 7
Details	58	91	submission	PTS	6-pyruvoyltetrahydropterin synthase
Details	59	91	submission	GRIK2	glutamate ionotropic receptor kainate type subunit 2
Details	60	91	submission	FZD1	frizzled class receptor 1
Details	61	91	submission	ELL2	elongation factor for RNA polymerase II 2
Details	62	91	submission	PRKAA2	protein kinase AMP-activated catalytic subunit alpha 2
Details	63	91	submission	RORA	RAR related orphan receptor A

Details	64	91	submission	RAI2	retinoic acid induced 2
Details	65	91	submission	POU3F2	POU class 3 homeobox 2
Details	66	91	submission	VAPB	VAMP associated protein B and C
Details	67	91	submission	VXN	vexin
Details	68	90	submission	CLGN	calmegin
Details	69	90	submission	OMD	osteomodulin
Details	70	90	submission	ZNF697	zinc finger protein 697
Details	71	90	submission	DEFB134	defensin beta 134
Details	72	90	submission	ABR	ABR, RhoGEF and GTPase activating protein
Details	73	90	submission	GNA13	G protein subunit alpha 13
Details	74	90	submission	BIVM	basic, immunoglobulin-like variable motif containing
Details	75	90	submission	CREB5	cAMP responsive element binding protein 5
Details	76	90	submission	ROR1	receptor tyrosine kinase like orphan receptor 1
Details	77	90	submission	DNAJC28	DnaJ heat shock protein family (Hsp40) member C28
Details	78	90	submission	HYKK	hydroxylysine kinase
Details	79	89	submission	SCNM1	sodium channel modifier 1
Details	80	89	submission	TNFAIP8L2-SCNM1	TNFAIP8L2-SCNM1 readthrough
Details	81	89	submission	SRGAP1	SLIT-ROBO Rho GTPase activating protein 1
Details	82	89	submission	SLC7A11	solute carrier family 7 member 11
Details	83	89	submission	RAB23	RAB23, member RAS oncogene family
Details	84	89	submission	ANGPTL7	angiopoietin like 7
Details	85	89	submission	SGO1	shugoshin 1
Details	86	89	submission	TMEM70	transmembrane protein 70
Details	87	89	submission	MEIS2	Meis homeobox 2
Details	88	89	submission	ZFHX3	zinc finger homeobox 3
Details	89	89	submission	CDCA2	cell division cycle associated 2
Details	90	89	submission	ZBTB7A	zinc finger and BTB domain containing 7A
Details	91	88	submission	ACER3	alkaline ceramidase 3
Details	92	88	submission	IDH3A	isocitrate dehydrogenase 3 (NAD(+)) alpha
Details	93	88	submission	ICOS	inducible T cell costimulator
Details	94	88	submission	FMNL3	formin like 3
Details	95	88	submission	AMDHD1	amidohydrolase domain containing 1
Details	96	88	submission	EPB41L3	erythrocyte membrane protein band 4.1 like 3
Details	97	88	submission	RBL2	RB transcriptional corepressor like 2
Details	98	88	submission	ZNF720	zinc finger protein 720
Details	99	88	submission	SPDYA	speedy/RINGO cell cycle regulator family member A

Details	100	88	submission	TSLP	thymic stromal lymphopoietin
Details	101	88	submission	ENPP6	ectonucleotide pyrophosphatase/phosphodiesterase 6
Details	102	88	submission	MYSM1	Myb like, SWIRM and MPN domains 1
Details	103	88	submission	GRM8	glutamate metabotropic receptor 8
Details	104	87	submission	ATG12	autophagy related 12
Details	105	87	submission	ADD1	adducin 1
Details	106	87	submission	RNF113B	ring finger protein 113B
Details	107	87	submission	C1orf198	chromosome 1 open reading frame 198
Details	108	87	submission	USP9Y	ubiquitin specific peptidase 9 Y-linked
Details	109	87	submission	PLCL1	phospholipase C like 1 (inactive)
Details	110	87	submission	TFDP1	transcription factor Dp-1
Details	111	87	submission	UGT2A3	UDP glucuronosyltransferase family 2 member A3
Details	112	87	submission	NTF4	neurotrophin 4
Details	113	87	submission	PKD2	polycystin 2, transient receptor potential cation channel
Details	114	87	submission	NRXN1	neurexin 1
Details	115	87	submission	B4GALT5	beta-1,4-galactosyltransferase 5
Details	116	87	submission	PMEPA1	prostate transmembrane protein, androgen induced 1
Details	117	87	submission	ATG4C	autophagy related 4C cysteine peptidase
Details	118	87	submission	KLHL7	kelch like family member 7
Details	119	87	submission	RBM26	RNA binding motif protein 26
Details	120	87	submission	UBXN2B	UBX domain protein 2B
Details	121	87	submission	SEMA5A	semaphorin 5A
Details	122	87	submission	ACAP2	ArfGAP with coiled-coil, ankyrin repeat and PH domains 2
Details	123	86	submission	HDAC8	histone deacetylase 8
Details	124	86	submission	BAALC	BAALC, MAP3K1 and KLF4 binding
Details	125	86	submission	EXOSC9	exosome component 9
Details	126	86	submission	FAAP20	FA core complex associated protein 20
Details	127	86	submission	USP44	ubiquitin specific peptidase 44
Details	128	86	submission	CYP27B1	cytochrome P450 family 27 subfamily B member 1
Details	129	86	submission	LMNTD1	lamin tail domain containing 1
Details	130	86	submission	CELSR3	cadherin EGF LAG seven-pass G-type receptor 3
Details	131	86	submission	SPEG	striated muscle enriched protein kinase
Details	132	86	submission	CRNKL1	crooked neck pre-mRNA splicing factor 1
Details	133	86	submission	TEX2	testis expressed 2
Details	134	86	submission	APAF1	apoptotic peptidase activating factor 1
Details	135	86	submission	HIPK3	homeodomain interacting protein kinase 3

Details	136	86	submission	HSD17B13	hydroxysteroid 17-beta dehydrogenase 13
Details	137	86	submission	RASA3	RAS p21 protein activator 3
Details	138	86	submission	RNF139	ring finger protein 139
Details	139	86	submission	KCNMA1	potassium calcium-activated channel subfamily M alpha 1
Details	140	85	submission	UST	uronyl 2-sulfotransferase
Details	141	85	submission	LRP2BP	LRP2 binding protein
Details	142	85	submission	TCF7L1	transcription factor 7 like 1
Details	143	85	submission	SYNJ1	synaptojanin 1
Details	144	85	submission	YIPF4	Yip1 domain family member 4
Details	145	85	submission	ABHD10	abhydrolase domain containing 10
Details	146	85	submission	KAT6A	lysine acetyltransferase 6A
Details	147	85	submission	PHC3	polyhomeotic homolog 3
Details	148	85	submission	ZNF454	zinc finger protein 454
Details	149	85	submission	FMR1	fragile X mental retardation 1
Details	150	85	submission	KRTAP4-8	keratin associated protein 4-8
Details	151	85	submission	KRTAP4-11	keratin associated protein 4-11
Details	152	85	submission	GTDC1	glycosyltransferase like domain containing 1
Details	153	85	submission	SYNCRIP	synaptotagmin binding cytoplasmic RNA interacting protein
Details	154	85	submission	MASP1	mannan binding lectin serine peptidase 1
Details	155	85	submission	TNRC6B	trinucleotide repeat containing 6B
Details	156	85	submission	ARGFX	arginine-fifty homeobox
Details	157	85	submission	TRIM35	tripartite motif containing 35
Details	158	84	submission	MSR1	macrophage scavenger receptor 1
Details	159	84	submission	WIPF1	WAS/WASL interacting protein family member 1
Details	160	84	submission	ZBTB41	zinc finger and BTB domain containing 41
Details	161	84	submission	MFAP3	microfibril associated protein 3
Details	162	84	submission	FYB2	FYN binding protein 2
Details	163	84	submission	BLZF1	basic leucine zipper nuclear factor 1
Details	164	84	submission	GAL3ST3	galactose-3-O-sulfotransferase 3
Details	165	84	submission	MIPOL1	mirror-image polydactyly 1
Details	166	84	submission	SGCD	sarcoglycan delta
Details	167	84	submission	MINDY3	MINDY lysine 48 deubiquitinase 3
Details	168	84	submission	ANKRD34B	ankyrin repeat domain 34B
Details	169	84	submission	WEE1	WEE1 G2 checkpoint kinase
Details	170	84	submission	NUCKS1	nuclear casein kinase and cyclin dependent kinase substrate 1
Details	171	84	submission	SLC39A6	solute carrier family 39 member 6

Details	172	84	submission	SERPINB10	serpin family B member 10
Details	173	84	submission	ZDBF2	zinc finger DBF-type containing 2
Details	174	84	submission	KCNJ15	potassium voltage-gated channel subfamily J member 15
Details	175	84	submission	COL4A1	collagen type IV alpha 1 chain
Details	176	84	submission	COX20	cytochrome c oxidase assembly factor COX20
Details	177	84	submission	PRRG1	proline rich and Gla domain 1
Details	178	83	submission	TBX4	T-box 4
Details	179	83	submission	RFX7	regulatory factor X7
Details	180	83	submission	KLHL29	kelch like family member 29
Details	181	83	submission	RHOBTB1	Rho related BTB domain containing 1
Details	182	83	submission	MAN2B2	mannosidase alpha class 2B member 2
Details	183	83	submission	PROX2	prospero homeobox 2
Details	184	83	submission	KDM3B	lysine demethylase 3B
Details	185	83	submission	TMEM123	transmembrane protein 123
Details	186	83	submission	IPO8	importin 8
Details	187	83	submission	IFT52	intraflagellar transport 52
Details	188	83	submission	MOB3B	MOB kinase activator 3B
Details	189	83	submission	PSMD5	proteasome 26S subunit, non-ATPase 5
Details	190	83	submission	SPARC	secreted protein acidic and cysteine rich
Details	191	83	submission	PALM2-AKAP2	PALM2-AKAP2 readthrough
Details	192	83	submission	AKAP2	A-kinase anchoring protein 2
Details	193	83	submission	ANK2	ankyrin 2
Details	194	83	submission	KLRC4	killer cell lectin like receptor C4
Details	195	83	submission	TCEAL7	transcription elongation factor A like 7
Details	196	83	submission	SCAI	suppressor of cancer cell invasion
Details	197	83	submission	EXOC2	exocyst complex component 2
Details	198	82	submission	NHLRC2	NHL repeat containing 2
Details	199	82	submission	SHCBP1L	SHC binding and spindle associated 1 like
Details	200	82	submission	ENPP4	ectonucleotide pyrophosphatase/phosphodiesterase 4
Details	201	82	submission	SLC47A2	solute carrier family 47 member 2
Details	202	82	submission	SORBS1	sorbin and SH3 domain containing 1
Details	203	82	submission	TNNI1	troponin I1, slow skeletal type
Details	204	82	submission	RIPOR2	RHO family interacting cell polarization regulator 2
Details	205	82	submission	BTBD1	BTB domain containing 1
Details	206	82	submission	MEGF11	multiple EGF like domains 11
Details	207	82	submission	RPL15	ribosomal protein L15
Details	208	82	submission	PDK1	pyruvate dehydrogenase kinase 1

Details	209	82	submission	ZFHX4	zinc finger homeobox 4
Details	210	81	submission	HOOK3	hook microtubule tethering protein 3
Details	211	81	submission	LIN9	lin-9 DREAM MuvB core complex component
Details	212	81	submission	CEP85L	centrosomal protein 85 like
Details	213	81	submission	ZNF689	zinc finger protein 689
Details	214	81	submission	PAQR8	progestin and adipoQ receptor family member 8
Details	215	81	submission	YTHDF2	YTH N6-methyladenosine RNA binding protein 2
Details	216	81	submission	RAB28	RAB28, member RAS oncogene family
Details	217	81	submission	EMSY	EMSY, BRCA2 interacting transcriptional repressor
Details	218	81	submission	TRIM2	tripartite motif containing 2
Details	219	81	submission	ADAMTS2	ADAM metallopeptidase with thrombospondin type 1 motif 2
Details	220	81	submission	HMGA1	high mobility group AT-hook 1
Details	221	81	submission	USP48	ubiquitin specific peptidase 48
Details	222	81	submission	KNCN	kinocilin
Details	223	81	submission	SORT1	sortilin 1
Details	224	81	submission	NADK2	NAD kinase 2, mitochondrial
Details	225	81	submission	GDA	guanine deaminase
Details	226	81	submission	CAMK4	calcium/calmodulin dependent protein kinase IV
Details	227	81	submission	PARP12	poly(ADP-ribose) polymerase family member 12
Details	228	81	submission	MBTD1	mbt domain containing 1
Details	229	80	submission	SUCNR1	succinate receptor 1
Details	230	80	submission	CLN3	CLN3, battenin
Details	231	80	submission	SLC35D1	solute carrier family 35 member D1
Details	232	80	submission	MYH2	myosin heavy chain 2
Details	233	80	submission	LRP8	LDL receptor related protein 8
Details	234	80	submission	DYNC2H1	dynein cytoplasmic 2 heavy chain 1
Details	235	80	submission	PLPPR1	phospholipid phosphatase related 1
Details	236	80	submission	ZNF292	zinc finger protein 292
Details	237	80	submission	SULF2	sulfatase 2
Details	238	80	submission	SUCLG2	succinate-CoA ligase GDP-forming beta subunit
Details	239	80	submission	ZNF106	zinc finger protein 106
Details	240	79	submission	NTM	neurotrimin
Details	241	79	submission	PCBD2	pterin-4 alpha-carbinolamine dehydratase 2
Details	242	79	submission	ABHD18	abhydrolase domain containing 18
Details	243	79	submission	PCYOX1	prenylcysteine oxidase 1

Details	244	79	submission	RWDD4	RWD domain containing 4
Details	245	79	submission	DCC	DCC netrin 1 receptor
Details	246	79	submission	SLC26A7	solute carrier family 26 member 7
Details	247	79	submission	LRFN2	leucine rich repeat and fibronectin type III domain containing 2
Details	248	79	submission	ZFP30	ZFP30 zinc finger protein
Details	249	79	submission	FGL2	fibrinogen like 2
Details	250	79	submission	DMXL1	Dmx like 1
Details	251	79	submission	CSNK1G1	casein kinase 1 gamma 1
Details	252	79	submission	TP53I11	tumor protein p53 inducible protein 11
Details	253	79	submission	STK40	serine/threonine kinase 40
Details	254	79	submission	MUC17	mucin 17, cell surface associated
Details	255	79	submission	STK32C	serine/threonine kinase 32C
Details	256	79	submission	PPP1R11	protein phosphatase 1 regulatory inhibitor subunit 11
Details	257	79	submission	ZFP1	ZFP1 zinc finger protein
Details	258	79	submission	NSUN4	NOP2/Sun RNA methyltransferase family member 4
Details	259	79	submission	HOXD10	homeobox D10
Details	260	78	submission	CORIN	corin, serine peptidase
Details	261	78	submission	MYO5B	myosin VB
Details	262	78	submission	CA10	carbonic anhydrase 10
Details	263	78	submission	RICTOR	RPTOR independent companion of MTOR complex 2
Details	264	78	submission	TDRP	testis development related protein
Details	265	78	submission	RIC1	RIC1 homolog, RAB6A GEF complex partner 1
Details	266	78	submission	ZBTB10	zinc finger and BTB domain containing 10
Details	267	78	submission	SLC25A24	solute carrier family 25 member 24
Details	268	78	submission	WDR17	WD repeat domain 17
Details	269	78	submission	METTL4	methyltransferase like 4
Details	270	78	submission	ZNF468	zinc finger protein 468
Details	271	78	submission	COMMD2	COMM domain containing 2
Details	272	78	submission	MAP10	microtubule associated protein 10
Details	273	78	submission	RNF217	ring finger protein 217
Details	274	78	submission	ABHD6	abhydrolase domain containing 6
Details	275	78	submission	GPR12	G protein-coupled receptor 12
Details	276	78	submission	LRIG1	leucine rich repeats and immunoglobulin like domains 1
Details	277	78	submission	GRAMD1C	GRAM domain containing 1C
Details	278	77	submission	QKI	QKI, KH domain containing RNA binding
Details	279	77	submission	ZNF554	zinc finger protein 554

Details	280	77	submission	CBL	Cbl proto-oncogene
Details	281	77	submission	DLG1	discs large MAGUK scaffold protein 1
Details	282	77	submission	FGF7	fibroblast growth factor 7
Details	283	77	submission	HDAC4	histone deacetylase 4
Details	284	77	submission	TRIM33	tripartite motif containing 33
Details	285	77	submission	COL6A5	collagen type VI alpha 5 chain
Details	286	77	submission	PRH2	proline rich protein HaeIII subfamily 2
Details	287	77	submission	RTL3	retrotransposon Gag like 3
Details	288	77	submission	XYLT1	xylosyltransferase 1
Details	289	77	submission	LYRM7	LYR motif containing 7
Details	290	77	submission	ZNF174	zinc finger protein 174
Details	291	77	submission	SRRM4	serine/arginine repetitive matrix 4
Details	292	76	submission	ERO1B	endoplasmic reticulum oxidoreductase 1 beta
Details	293	76	submission	CASTOR2	cytosolic arginine sensor for mTORC1 subunit 2
Details	294	76	submission	TPM1	tropomyosin 1
Details	295	76	submission	ZNF385A	zinc finger protein 385A
Details	296	76	submission	C7orf31	chromosome 7 open reading frame 31
Details	297	76	submission	TRIP11	thyroid hormone receptor interactor 11
Details	298	76	submission	CAMK1D	calcium/calmodulin dependent protein kinase ID
Details	299	76	submission	SLC39A10	solute carrier family 39 member 10
Details	300	76	submission	TTC14	tetratricopeptide repeat domain 14
Details	301	76	submission	CDC14B	cell division cycle 14B
Details	302	76	submission	TNPO1	transportin 1
Details	303	76	submission	FAM122C	family with sequence similarity 122C
Details	304	76	submission	FKTN	fukutin
Details	305	76	submission	TENM4	teneurin transmembrane protein 4
Details	306	76	submission	RBFOX3	RNA binding fox-1 homolog 3
Details	307	76	submission	SRSF10	serine and arginine rich splicing factor 10
Details	308	76	submission	OSBPL1A	oxysterol binding protein like 1A
Details	309	76	submission	SMIM13	small integral membrane protein 13
Details	310	76	submission	LIG4	DNA ligase 4
Details	311	76	submission	RBPMS	RNA binding protein, mRNA processing factor
Details	312	76	submission	GRM3	glutamate metabotropic receptor 3
Details	313	76	submission	NGDN	neuroguidin
Details	314	75	submission	ZNF75D	zinc finger protein 75D
Details	315	75	submission	DNAJC25	DnaJ heat shock protein family (Hsp40) member C25

Details	316	75	submission	ZNF699	zinc finger protein 699
Details	317	75	submission	ACSL1	acyl-CoA synthetase long chain family member 1
Details	318	75	submission	SNX29	sorting nexin 29
Details	319	75	submission	RFX3	regulatory factor X3
Details	320	75	submission	PIGA	phosphatidylinositol glycan anchor biosynthesis class A
Details	321	75	submission	GRIP1	glutamate receptor interacting protein 1
Details	322	75	submission	SYT4	synaptotagmin 4
Details	323	75	submission	USP32	ubiquitin specific peptidase 32
Details	324	75	submission	MSL1	MSL complex subunit 1
Details	325	75	submission	BMPR1B	bone morphogenetic protein receptor type 1B
Details	326	75	submission	IRS2	insulin receptor substrate 2
Details	327	75	submission	PRSS12	serine protease 12
Details	328	75	submission	XRCC2	X-ray repair cross complementing 2
Details	329	75	submission	NREP	neuronal regeneration related protein
Details	330	75	submission	DAPP1	dual adaptor of phosphotyrosine and 3-phosphoinositides 1
Details	331	75	submission	CTBP2	C-terminal binding protein 2
Details	332	75	submission	RBM12B	RNA binding motif protein 12B
Details	333	75	submission	SLC7A14	solute carrier family 7 member 14
Details	334	75	submission	NELL2	neural EGFL like 2
Details	335	75	submission	GRHL1	grainyhead like transcription factor 1
Details	336	75	submission	RPS6KA5	ribosomal protein S6 kinase A5
Details	337	75	submission	ZBTB24	zinc finger and BTB domain containing 24
Details	338	75	submission	RASA1	RAS p21 protein activator 1
Details	339	75	submission	PRKAR1A	protein kinase cAMP-dependent type I regulatory subunit alpha
Details	340	74	submission	ZFX	zinc finger protein X-linked
Details	341	74	submission	KCNK13	potassium two pore domain channel subfamily K member 13
Details	342	74	submission	NDC1	NDC1 transmembrane nucleoporin
Details	343	74	submission	ETFDH	electron transfer flavoprotein dehydrogenase
Details	344	74	submission	EFR3A	EFR3 homolog A
Details	345	74	submission	RNF114	ring finger protein 114
Details	346	74	submission	PLP2	proteolipid protein 2
Details	347	74	submission	HTRA3	HtrA serine peptidase 3
Details	348	74	submission	TRMT5	tRNA methyltransferase 5
Details	349	74	submission	TMX3	thioredoxin related transmembrane protein 3
Details	350	74	submission	ADCY1	adenylate cyclase 1

Details	351	74	submission	FBRSL1	fibrosin like 1
Details	352	74	submission	OTOP2	otopetrin 2
Details	353	74	submission	GNG4	G protein subunit gamma 4
Details	354	74	submission	GRM1	glutamate metabotropic receptor 1
Details	355	74	submission	MID2	midline 2
Details	356	74	submission	MEX3C	mex-3 RNA binding family member C
Details	357	74	submission	PCDHB4	protocadherin beta 4
Details	358	74	submission	ABCA1	ATP binding cassette subfamily A member 1
Details	359	74	submission	ENY2	ENY2, transcription and export complex 2 subunit
Details	360	73	submission	MEI4	meiotic double-stranded break formation protein 4
Details	361	73	submission	SLC22A23	solute carrier family 22 member 23
Details	362	73	submission	FAM20B	FAM20B, glycosaminoglycan xylosylkinase
Details	363	73	submission	BMP3	bone morphogenetic protein 3
Details	364	73	submission	PROK2	prokineticin 2
Details	365	73	submission	FBN2	fibrillin 2
Details	366	73	submission	UBQLN2	ubiquilin 2
Details	367	73	submission	DNASE2B	deoxyribonuclease 2 beta
Details	368	73	submission	MEF2C	myocyte enhancer factor 2C
Details	369	73	submission	NRG4	neuregulin 4
Details	370	73	submission	RXFP1	relaxin family peptide receptor 1
Details	371	73	submission	RPRD1A	regulation of nuclear pre-mRNA domain containing 1A
Details	372	73	submission	DLGAP4	DLG associated protein 4
Details	373	73	submission	NAV1	neuron navigator 1
Details	374	73	submission	SGMS2	sphingomyelin synthase 2
Details	375	73	submission	MS4A2	membrane spanning 4-domains A2
Details	376	72	submission	CKAP2L	cytoskeleton associated protein 2 like
Details	377	72	submission	ADGRG4	adhesion G protein-coupled receptor G4
Details	378	72	submission	NSMCE4A	NSE4 homolog A, SMC5-SMC6 complex component
Details	379	72	submission	PMP2	peripheral myelin protein 2
Details	380	72	submission	ZNF451	zinc finger protein 451
Details	381	72	submission	TRIM32	tripartite motif containing 32
Details	382	72	submission	NDN	necdin, MAGE family member
Details	383	72	submission	C10orf53	chromosome 10 open reading frame 53
Details	384	72	submission	HEY1	hes related family bHLH transcription factor with YRPW motif 1
Details	385	71	submission	PAK3	p21 (RAC1) activated kinase 3
Details	386	71	submission	ATG5	autophagy related 5

Details	387	71	submission	FLRT2	fibronectin leucine rich transmembrane protein 2
Details	388	71	submission	HECTD2	HECT domain E3 ubiquitin protein ligase 2
Details	389	71	submission	LSAMP	limbic system associated membrane protein
Details	390	71	submission	LNX1	ligand of numb-protein X 1
Details	391	71	submission	CDH11	cadherin 11
Details	392	71	submission	ZADH2	zinc binding alcohol dehydrogenase domain containing 2
Details	393	71	submission	ZDHHC17	zinc finger DHHC-type containing 17
Details	394	71	submission	TOP3A	DNA topoisomerase III alpha
Details	395	71	submission	SKP2	S-phase kinase associated protein 2
Details	396	71	submission	U2SURP	U2 snRNP associated SURP domain containing
Details	397	71	submission	PAG1	phosphoprotein membrane anchor with glycosphingolipid microdomains 1
Details	398	71	submission	KHSRP	KH-type splicing regulatory protein
Details	399	71	submission	RYBP	RING1 and YY1 binding protein
Details	400	70	submission	FAM241A	family with sequence similarity 241 member A
Details	401	70	submission	CDKN1B	cyclin dependent kinase inhibitor 1B
Details	402	70	submission	RPS6KA3	ribosomal protein S6 kinase A3
Details	403	70	submission	PIK3R1	phosphoinositide-3-kinase regulatory subunit 1
Details	404	70	submission	IGFBP3	insulin like growth factor binding protein 3
Details	405	70	submission	FBXL20	F-box and leucine rich repeat protein 20
Details	406	70	submission	PEX5L	peroxisomal biogenesis factor 5 like
Details	407	70	submission	BDP1	B double prime 1, subunit of RNA polymerase III transcription initiation factor IIIB
Details	408	70	submission	KHDC4	KH domain containing 4, pre-mRNA splicing factor
Details	409	70	submission	CREBZF	CREB/ATF bZIP transcription factor
Details	410	70	submission	CTBS	chitobiase
Details	411	70	submission	BNIP3L	BCL2 interacting protein 3 like
Details	412	70	submission	CHUK	conserved helix-loop-helix ubiquitous kinase
Details	413	69	submission	RNF6	ring finger protein 6
Details	414	69	submission	IL6ST	interleukin 6 signal transducer
Details	415	69	submission	FBXO33	F-box protein 33
Details	416	69	submission	MCM3	minichromosome maintenance complex component 3
Details	417	69	submission	WDR35	WD repeat domain 35
Details	418	69	submission	TUBGCP3	tubulin gamma complex associated protein 3
Details	419	69	submission	GPR137C	G protein-coupled receptor 137C

Details	420	69	submission	CDC27	cell division cycle 27
Details	421	69	submission	COL4A4	collagen type IV alpha 4 chain
Details	422	69	submission	ZSWIM6	zinc finger SWIM-type containing 6
Details	423	69	submission	BCAS2	BCAS2, pre-mRNA processing factor
Details	424	69	submission	NUDT12	nudix hydrolase 12
Details	425	69	submission	ATAT1	alpha tubulin acetyltransferase 1
Details	426	69	submission	ITGAV	integrin subunit alpha V
Details	427	69	submission	CCDC170	coiled-coil domain containing 170
Details	428	69	submission	CRCP	CGRP receptor component
Details	429	69	submission	ZNF615	zinc finger protein 615
Details	430	68	submission	FOXR2	forkhead box R2
Details	431	68	submission	SMLR1	small leucine rich protein 1
Details	432	68	submission	SNX30	sorting nexin family member 30
Details	433	68	submission	EIF3A	eukaryotic translation initiation factor 3 subunit A
Details	434	68	submission	C2CD4A	C2 calcium dependent domain containing 4A
Details	435	68	submission	HEPACAM2	HEPACAM family member 2
Details	436	68	submission	ADRB1	adrenoceptor beta 1
Details	437	68	submission	BCL2L1	BCL2 like 1
Details	438	68	submission	KIRREL1	kirre like nephrin family adhesion molecule 1
Details	439	68	submission	NR3C2	nuclear receptor subfamily 3 group C member 2
Details	440	68	submission	PAXIP1	PAX interacting protein 1
Details	441	68	submission	METTL15	methyltransferase like 15
Details	442	68	submission	NR1I2	nuclear receptor subfamily 1 group I member 2
Details	443	68	submission	JMY	junction mediating and regulatory protein, p53 cofactor
Details	444	67	submission	QSER1	glutamine and serine rich 1
Details	445	67	submission	ARHGAP6	Rho GTPase activating protein 6
Details	446	67	submission	TRIM5	tripartite motif containing 5
Details	447	67	submission	BRAP	BRCA1 associated protein
Details	448	67	submission	TMC7	transmembrane channel like 7
Details	449	67	submission	PTPRM	protein tyrosine phosphatase, receptor type M
Details	450	67	submission	ARID4B	AT-rich interaction domain 4B
Details	451	67	submission	VPS37A	VPS37A, ESCRT-I subunit
Details	452	67	submission	ZNF704	zinc finger protein 704
Details	453	67	submission	DACT3	dishevelled binding antagonist of beta catenin 3
Details	454	67	submission	EIF4G2	eukaryotic translation initiation factor 4 gamma 2

Details	455	67	submission	NUBPL	nucleotide binding protein like
Details	456	67	submission	MINDY2	MINDY lysine 48 deubiquitinase 2
Details	457	67	submission	SSBP2	single stranded DNA binding protein 2
Details	458	67	submission	TNFRSF11A	TNF receptor superfamily member 11a
Details	459	67	submission	CNNM4	cyclin and CBS domain divalent metal cation transport mediator 4
Details	460	67	submission	CTPS2	CTP synthase 2
Details	461	67	submission	TSPY10	testis specific protein Y-linked 10
Details	462	67	submission	KCNQ5	potassium voltage-gated channel subfamily Q member 5
Details	463	66	submission	LEPROTL1	leptin receptor overlapping transcript like 1
Details	464	66	submission	STXBP5L	syntaxin binding protein 5 like
Details	465	66	submission	PTPN14	protein tyrosine phosphatase, non-receptor type 14
Details	466	66	submission	ANO2	anoctamin 2
Details	467	66	submission	ZEB2	zinc finger E-box binding homeobox 2
Details	468	66	submission	ZNF709	zinc finger protein 709
Details	469	66	submission	KIAA1549	KIAA1549
Details	470	66	submission	OTULINL	OTU deubiquitinase with linear linkage specificity like
Details	471	66	submission	TYW3	tRNA-yW synthesizing protein 3 homolog
Details	472	66	submission	CDH19	cadherin 19
Details	473	66	submission	USP10	ubiquitin specific peptidase 10
Details	474	66	submission	LMAN1	lectin, mannose binding 1
Details	475	66	submission	MYH1	myosin heavy chain 1
Details	476	66	submission	ADO	2-aminoethanethiol dioxygenase
Details	477	66	submission	PURA	purine rich element binding protein A
Details	478	66	submission	PDIK1L	PDLIM1 interacting kinase 1 like
Details	479	66	submission	PRKG1	protein kinase cGMP-dependent 1
Details	480	66	submission	MPRIP	myosin phosphatase Rho interacting protein
Details	481	66	submission	DTNBP1	dystrobrevin binding protein 1
Details	482	65	submission	GRIP2	glutamate receptor interacting protein 2
Details	483	65	submission	SEMA6A	semaphorin 6A
Details	484	65	submission	TRIB1	tribbles pseudokinase 1
Details	485	65	submission	TET2	tet methylcytosine dioxygenase 2
Details	486	65	submission	PKN2	protein kinase N2
Details	487	65	submission	HOMER1	homer scaffold protein 1
Details	488	65	submission	PITPNB	phosphatidylinositol transfer protein beta
Details	489	65	submission	UGT2B4	UDP glucuronosyltransferase family 2 member B4
Details	490	65	submission	ZDHHC21	zinc finger DHHC-type containing 21

Details	491	65	submission	EBF1	EBF transcription factor 1
Details	492	65	submission	TMEM9B	TMEM9 domain family member B
Details	493	65	submission	KDM6A	lysine demethylase 6A
Details	494	65	submission	UBE4A	ubiquitination factor E4A
Details	495	65	submission	LACC1	laccase domain containing 1
Details	496	65	submission	ZNF618	zinc finger protein 618
Details	497	65	submission	SYT13	synaptotagmin 13
Details	498	65	submission	MSH4	mutS homolog 4
Details	499	65	submission	STAT5B	signal transducer and activator of transcription 5B
Details	500	65	submission	ZNF28	zinc finger protein 28
Details	501	65	submission	CCR6	C-C motif chemokine receptor 6
Details	502	65	submission	CAMK2A	calcium/calmodulin dependent protein kinase II alpha
Details	503	65	submission	LIN7A	lin-7 homolog A, crumbs cell polarity complex component
Details	504	65	submission	CFDP1	craniofacial development protein 1
Details	505	64	submission	ITGBL1	integrin subunit beta like 1
Details	506	64	submission	SPATA31D4	SPATA31 subfamily D member 4
Details	507	64	submission	SEPT3	septin 3
Details	508	64	submission	NCOA2	nuclear receptor coactivator 2
Details	509	64	submission	INSR	insulin receptor
Details	510	64	submission	ADARB2	adenosine deaminase, RNA specific B2 (inactive)
Details	511	64	submission	C6orf120	chromosome 6 open reading frame 120
Details	512	64	submission	PLD5	phospholipase D family member 5
Details	513	64	submission	EID1	EP300 interacting inhibitor of differentiation 1
Details	514	64	submission	RAB29	RAB29, member RAS oncogene family
Details	515	64	submission	TRIM37	tripartite motif containing 37
Details	516	64	submission	SHISAL1	shisa like 1
Details	517	64	submission	IRAK3	interleukin 1 receptor associated kinase 3
Details	518	64	submission	APLF	aprataxin and PNKP like factor
Details	519	64	submission	COL4A3BP	collagen type IV alpha 3 binding protein
Details	520	64	submission	MED12L	mediator complex subunit 12 like
Details	521	64	submission	KRIT1	KRIT1, ankyrin repeat containing
Details	522	64	submission	ZNF781	zinc finger protein 781
Details	523	64	submission	CYP4F3	cytochrome P450 family 4 subfamily F member 3
Details	524	64	submission	HSPA13	heat shock protein family A (Hsp70) member 13
Details	525	64	submission	CASK	calcium/calmodulin dependent serine protein kinase

Details	526	64	submission	PROX1	prospero homeobox 1
Details	527	64	submission	TBC1D26	TBC1 domain family member 26
Details	528	64	submission	CDK15	cyclin dependent kinase 15
Details	529	64	submission	SOCS7	suppressor of cytokine signaling 7
Details	530	64	submission	IFNLR1	interferon lambda receptor 1
Details	531	63	submission	LIFR	LIF receptor alpha
Details	532	63	submission	ZNF521	zinc finger protein 521
Details	533	63	submission	TIAM2	T cell lymphoma invasion and metastasis 2
Details	534	63	submission	TMED5	transmembrane p24 trafficking protein 5
Details	535	63	submission	RIMS2	regulating synaptic membrane exocytosis 2
Details	536	63	submission	C3orf70	chromosome 3 open reading frame 70
Details	537	63	submission	CENPP	centromere protein P
Details	538	63	submission	THSD7A	thrombospondin type 1 domain containing 7A
Details	539	63	submission	PURG	purine rich element binding protein G
Details	540	63	submission	SVOP	SV2 related protein
Details	541	63	submission	TIGIT	T cell immunoreceptor with Ig and ITIM domains
Details	542	63	submission	LOC100144595	uncharacterized LOC100144595
Details	543	63	submission	TMX4	thioredoxin related transmembrane protein 4
Details	544	63	submission	GDNF	glial cell derived neurotrophic factor
Details	545	63	submission	PKIA	cAMP-dependent protein kinase inhibitor alpha
Details	546	63	submission	COG6	component of oligomeric golgi complex 6
Details	547	63	submission	TSPY2	testis specific protein Y-linked 2
Details	548	63	submission	TSPY1	testis specific protein Y-linked 1
Details	549	63	submission	TSPY8	testis specific protein Y-linked 8
Details	550	63	submission	TSPY4	testis specific protein Y-linked 4
Details	551	63	submission	TSPY3	testis specific protein Y-linked 3
Details	552	63	submission	ZNF470	zinc finger protein 470
Details	553	63	submission	RAB9A	RAB9A, member RAS oncogene family
Details	554	63	submission	GINS3	GINS complex subunit 3
Details	555	63	submission	SBF2	SET binding factor 2
Details	556	63	submission	MALT1	MALT1 paracaspase
Details	557	63	submission	BTLA	B and T lymphocyte associated
Details	558	63	submission	IKZF2	IKAROS family zinc finger 2
Details	559	63	submission	HUNK	hormonally up-regulated Neu-associated kinase
Details	560	63	submission	PSAPL1	prosaposin like 1 (gene/pseudogene)
Details	561	63	submission	GGACT	gamma-glutamylamine cyclotransferase

Details	562	63	submission	TRPC5OS	TRPC5 opposite strand
Details	563	63	submission	ZNF250	zinc finger protein 250
Details	564	63	submission	CRTAM	cytotoxic and regulatory T cell molecule
Details	565	63	submission	VCAM1	vascular cell adhesion molecule 1
Details	566	63	submission	ZNF18	zinc finger protein 18
Details	567	62	submission	ERC1	ELKS/RAB6-interacting/CAST family member 1
Details	568	62	submission	JAG1	jagged 1
Details	569	62	submission	CELF4	CUGBP Elav-like family member 4
Details	570	62	submission	EIF4A1	eukaryotic translation initiation factor 4A1
Details	571	62	submission	AGAP11	ArfGAP with GTPase domain, ankyrin repeat and PH domain 11
Details	572	62	submission	DCUN1D1	defective in cullin neddylation 1 domain containing 1
Details	573	62	submission	GABRG2	gamma-aminobutyric acid type A receptor gamma2 subunit
Details	574	62	submission	SLC25A17	solute carrier family 25 member 17
Details	575	62	submission	STAM2	signal transducing adaptor molecule 2
Details	576	62	submission	AGPAT5	1-acylglycerol-3-phosphate O-acyltransferase 5
Details	577	62	submission	GABBR2	gamma-aminobutyric acid type B receptor subunit 2
Details	578	62	submission	RELN	reelin
Details	579	62	submission	AGAP6	ArfGAP with GTPase domain, ankyrin repeat and PH domain 6
Details	580	62	submission	HIBCH	3-hydroxyisobutyryl-CoA hydrolase
Details	581	62	submission	UBN2	ubinuclein 2
Details	582	62	submission	AGAP4	ArfGAP with GTPase domain, ankyrin repeat and PH domain 4
Details	583	62	submission	SH3PXD2A	SH3 and PX domains 2A
Details	584	62	submission	AGAP5	ArfGAP with GTPase domain, ankyrin repeat and PH domain 5
Details	585	62	submission	HPCAL4	hippocalcin like 4
Details	586	62	submission	AGAP9	ArfGAP with GTPase domain, ankyrin repeat and PH domain 9
Details	587	62	submission	PTPN4	protein tyrosine phosphatase, non-receptor type 4
Details	588	61	submission	ARMC9	armadillo repeat containing 9
Details	589	61	submission	DGKE	diacylglycerol kinase epsilon
Details	590	61	submission	MFSD4A	major facilitator superfamily domain containing 4A
Details	591	61	submission	EPHA6	EPH receptor A6
Details	592	61	submission	ACAT2	acetyl-CoA acetyltransferase 2
Details	593	61	submission	TSHZ2	teashirt zinc finger homeobox 2
Details	594	61	submission	KIAA1522	KIAA1522

Details	595	61	submission	ALG1	ALG1, chitobiosyldiphosphodolichol beta-mannosyltransferase
Details	596	61	submission	TMEM59	transmembrane protein 59
Details	597	61	submission	RET	ret proto-oncogene
Details	598	61	submission	SHANK2	SH3 and multiple ankyrin repeat domains 2
Details	599	61	submission	GPC6	glypican 6
Details	600	61	submission	SLC2A12	solute carrier family 2 member 12
Details	601	61	submission	EDEM3	ER degradation enhancing alpha-mannosidase like protein 3
Details	602	61	submission	SMIM9	small integral membrane protein 9
Details	603	61	submission	EXTL2	exostosin like glycosyltransferase 2
Details	604	61	submission	CEBPG	CCAAT enhancer binding protein gamma
Details	605	61	submission	SWT1	SWT1, RNA endoribonuclease homolog
Details	606	61	submission	RASSF8	Ras association domain family member 8
Details	607	60	submission	PCDH9	protocadherin 9
Details	608	60	submission	TRMT13	tRNA methyltransferase 13 homolog
Details	609	60	submission	ZNF559-ZNF177	ZNF559-ZNF177 readthrough
Details	610	60	submission	RBM15B	RNA binding motif protein 15B
Details	611	60	submission	BICC1	BicC family RNA binding protein 1
Details	612	60	submission	DACT1	dishevelled binding antagonist of beta catenin 1
Details	613	60	submission	STRBP	spermatid perinuclear RNA binding protein
Details	614	60	submission	TCF4	transcription factor 4
Details	615	60	submission	SORCS1	sortilin related VPS10 domain containing receptor 1
Details	616	60	submission	ANOS1	anosmin 1
Details	617	60	submission	AMN1	antagonist of mitotic exit network 1 homolog
Details	618	60	submission	ANKRD36B	ankyrin repeat domain 36B
Details	619	60	submission	C1orf109	chromosome 1 open reading frame 109
Details	620	60	submission	IGF2BP2	insulin like growth factor 2 mRNA binding protein 2
Details	621	60	submission	ZNF561	zinc finger protein 561
Details	622	60	submission	PSTPIP2	proline-serine-threonine phosphatase interacting protein 2
Details	623	60	submission	KCNJ13	potassium voltage-gated channel subfamily J member 13
Details	624	60	submission	AIMP1	aminoacyl tRNA synthetase complex interacting multifunctional protein 1
Details	625	60	submission	CD209	CD209 molecule
Details	626	60	submission	MYH8	myosin heavy chain 8
Details	627	60	submission	ACTN4	actinin alpha 4
Details	628	59	submission	C16orf58	chromosome 16 open reading frame 58

Details	629	59	submission	CARMIL1	capping protein regulator and myosin 1 linker 1
Details	630	59	submission	CNTD2	cyclin N-terminal domain containing 2
Details	631	59	submission	VPS13C	vacuolar protein sorting 13 homolog C
Details	632	59	submission	FRMD5	FERM domain containing 5
Details	633	59	submission	MPP6	membrane palmitoylated protein 6
Details	634	59	submission	DPP4	dipeptidyl peptidase 4
Details	635	59	submission	ERC2	ELKS/RAB6-interacting/CAST family member 2
Details	636	59	submission	LZTS3	leucine zipper tumor suppressor family member 3
Details	637	59	submission	PTGFR	prostaglandin F receptor
Details	638	59	submission	ARRDC3	arrestin domain containing 3
Details	639	59	submission	OCLN	occludin
Details	640	59	submission	PUS7L	pseudouridine synthase 7 like
Details	641	59	submission	ZFHX2	zinc finger homeobox 2
Details	642	59	submission	RUBCNL	rubicon like autophagy enhancer
Details	643	59	submission	ZNF425	zinc finger protein 425
Details	644	59	submission	SYNPO2	synaptopodin 2
Details	645	59	submission	C1GALT1	core 1 synthase, glycoprotein-N-acetylgalactosamine 3-beta-galactosyltransferase 1
Details	646	59	submission	RBM41	RNA binding motif protein 41
Details	647	59	submission	ZNF678	zinc finger protein 678
Details	648	59	submission	TIPARP	TCDD inducible poly(ADP-ribose) polymerase
Details	649	58	submission	CACNB4	calcium voltage-gated channel auxiliary subunit beta 4
Details	650	58	submission	PRR27	proline rich 27
Details	651	58	submission	CBWD5	COBW domain containing 5
Details	652	58	submission	CBWD6	COBW domain containing 6
Details	653	58	submission	CERS6	ceramide synthase 6
Details	654	58	submission	BRCA1	BRCA1, DNA repair associated
Details	655	58	submission	CBWD1	COBW domain containing 1
Details	656	58	submission	RBBP4	RB binding protein 4, chromatin remodeling factor
Details	657	58	submission	XG	Xg glycoprotein (Xg blood group)
Details	658	58	submission	TBX5	T-box 5
Details	659	58	submission	KCTD14	potassium channel tetramerization domain containing 14
Details	660	58	submission	ST8SIA3	ST8 alpha-N-acetyl-neuraminide alpha-2,8-sialyltransferase 3
Details	661	58	submission	CBWD3	COBW domain containing 3
Details	662	58	submission	CCDC88A	coiled-coil domain containing 88A

Details	663	58	submission	YOD1	YOD1 deubiquitinase
Details	664	58	submission	ANKRD27	ankyrin repeat domain 27
Details	665	58	submission	ANXA10	annexin A10
Details	666	58	submission	PPM1L	protein phosphatase, Mg2+/Mn2+ dependent 1L
Details	667	58	submission	SRFBP1	serum response factor binding protein 1
Details	668	58	submission	FAM86C1	family with sequence similarity 86 member C1
Details	669	58	submission	TRAK2	trafficking kinesin protein 2
Details	670	58	submission	REPS2	RALBP1 associated Eps domain containing 2
Details	671	58	submission	POU2F2	POU class 2 homeobox 2
Details	672	58	submission	APOLD1	apolipoprotein L domain containing 1
Details	673	58	submission	CUX1	cut like homeobox 1
Details	674	58	submission	TSPYL4	TSPY like 4
Details	675	58	submission	CBWD2	COBW domain containing 2
Details	676	58	submission	CYBRD1	cytochrome b reductase 1
Details	677	58	submission	DDX5	DEAD-box helicase 5
Details	678	58	submission	GPATCH2L	G-patch domain containing 2 like
Details	679	58	submission	NABP1	nucleic acid binding protein 1
Details	680	57	submission	ST6GALNAC3	ST6 N-acetylgalactosaminide alpha-2,6-sialyltransferase 3
Details	681	57	submission	STYXL1	serine/threonine/tyrosine interacting like 1
Details	682	57	submission	RASGRP1	RAS guanyl releasing protein 1
Details	683	57	submission	RAB27B	RAB27B, member RAS oncogene family
Details	684	57	submission	PRG4	proteoglycan 4
Details	685	57	submission	LRRC1	leucine rich repeat containing 1
Details	686	57	submission	SESN3	sestrin 3
Details	687	57	submission	HLF	HLF, PAR bZIP transcription factor
Details	688	57	submission	AGAP1	ArfGAP with GTPase domain, ankyrin repeat and PH domain 1
Details	689	57	submission	CTSV	cathepsin V
Details	690	57	submission	ANK3	ankyrin 3
Details	691	57	submission	TMEFF1	transmembrane protein with EGF like and two follistatin like domains 1
Details	692	57	submission	MSANTD3-TMEFF1	MSANTD3-TMEFF1 readthrough
Details	693	57	submission	DNAJA4	DnaJ heat shock protein family (Hsp40) member A4
Details	694	57	submission	EBF2	EBF transcription factor 2
Details	695	57	submission	PCGF3	polycomb group ring finger 3
Details	696	57	submission	BROX	BRO1 domain and CAAX motif containing
Details	697	57	submission	SEC62	SEC62 homolog, preprotein translocation factor

Details	698	57	submission	SBSPON	somatomedin B and thrombospondin type 1 domain containing
Details	699	57	submission	NEXMIF	neurite extension and migration factor
Details	700	57	submission	USH2A	usherin
Details	701	57	submission	RNF152	ring finger protein 152
Details	702	57	submission	IL17RD	interleukin 17 receptor D
Details	703	57	submission	USP15	ubiquitin specific peptidase 15
Details	704	57	submission	CTTNBP2NL	CTTNBP2 N-terminal like
Details	705	57	submission	SUN2	Sad1 and UNC84 domain containing 2
Details	706	57	submission	ATXN1	ataxin 1
Details	707	57	submission	PIK3CA	phosphatidylinositol-4,5-bisphosphate 3-kinase catalytic subunit alpha
Details	708	57	submission	MB21D2	Mab-21 domain containing 2
Details	709	57	submission	SYNPR	synaptoporin
Details	710	57	submission	ATRN	attractin
Details	711	57	submission	EPHA7	EPH receptor A7
Details	712	57	submission	ITGA6	integrin subunit alpha 6
Details	713	57	submission	KIF5B	kinesin family member 5B
Details	714	57	submission	NTRK2	neurotrophic receptor tyrosine kinase 2
Details	715	57	submission	CLDN10	claudin 10
Details	716	57	submission	RPL22L1	ribosomal protein L22 like 1
Details	717	57	submission	APBA1	amyloid beta precursor protein binding family A member 1
Details	718	56	submission	PARP9	poly(ADP-ribose) polymerase family member 9
Details	719	56	submission	CUL3	cullin 3
Details	720	56	submission	PDLIM4	PDZ and LIM domain 4
Details	721	56	submission	SMPDL3B	sphingomyelin phosphodiesterase acid like 3B
Details	722	56	submission	PUDP	pseudouridine 5'-phosphatase
Details	723	56	submission	SIDT1	SID1 transmembrane family member 1
Details	724	56	submission	RBAK	RB associated KRAB zinc finger
Details	725	56	submission	TYRP1	tyrosinase related protein 1
Details	726	56	submission	FRMD3	FERM domain containing 3
Details	727	56	submission	MAGEE2	MAGE family member E2
Details	728	56	submission	INSC	INSC, spindle orientation adaptor protein
Details	729	56	submission	RASGRP4	RAS guanyl releasing protein 4
Details	730	56	submission	ADAMTS17	ADAM metallopeptidase with thrombospondin type 1 motif 17
Details	731	56	submission	SLC9A2	solute carrier family 9 member A2
Details	732	56	submission	SAMD8	sterile alpha motif domain containing 8
Details	733	56	submission	CDK19	cyclin dependent kinase 19

Details	734	56	submission	SMIM8	small integral membrane protein 8
Details	735	56	submission	GPATCH2	G-patch domain containing 2
Details	736	55	submission	E2F6	E2F transcription factor 6
Details	737	55	submission	SHMT1	serine hydroxymethyltransferase 1
Details	738	55	submission	ZNF714	zinc finger protein 714
Details	739	55	submission	UFL1	UFM1 specific ligase 1
Details	740	55	submission	FAM160A1	family with sequence similarity 160 member A1
Details	741	55	submission	ANKH	ANKH inorganic pyrophosphate transport regulator
Details	742	55	submission	DPPA4	developmental pluripotency associated 4
Details	743	55	submission	PATJ	PATJ, crumbs cell polarity complex component
Details	744	55	submission	SASH1	SAM and SH3 domain containing 1
Details	745	55	submission	PAPOLB	poly(A) polymerase beta
Details	746	55	submission	ZC3H12C	zinc finger CCCH-type containing 12C
Details	747	55	submission	PHF3	PHD finger protein 3
Details	748	55	submission	OPRM1	opioid receptor mu 1
Details	749	55	submission	MPC2	mitochondrial pyruvate carrier 2
Details	750	55	submission	KIF21B	kinesin family member 21B
Details	751	55	submission	AP2B1	adaptor related protein complex 2 subunit beta 1
Details	752	55	submission	GOLGA6C	golgin A6 family member C
Details	753	55	submission	ZMYM2	zinc finger MYM-type containing 2
Details	754	55	submission	PRH1-TAS2R14	PRH1-TAS2R14 readthrough
Details	755	55	submission	JADE1	jade family PHD finger 1
Details	756	55	submission	SRXN1	sulfiredoxin 1
Details	757	55	submission	CWC27	CWC27 spliceosome associated protein homolog
Details	758	55	submission	INVS	inversin
Details	759	55	submission	LRP1B	LDL receptor related protein 1B
Details	760	55	submission	CACNA1I	calcium voltage-gated channel subunit alpha1 I
Details	761	55	submission	C5orf63	chromosome 5 open reading frame 63
Details	762	55	submission	INAFM2	InaF motif containing 2
Details	763	55	submission	LYST	lysosomal trafficking regulator
Details	764	55	submission	SREK1IP1	SREK1 interacting protein 1
Details	765	55	submission	DNHD1	dynein heavy chain domain 1
Details	766	54	submission	BMPR2	bone morphogenetic protein receptor type 2
Details	767	54	submission	APLN	apelin

B) miR-MAK-403

miRDB

There are 665 predicted targets for the submitted 22 nt long miRNA: aucaagaggaagaauguacaac (miR-MAK-403)

Return to Custom Prediction

Target Detail	Target Rank	Target Score	miRNA Name	Gene Symbol	Gene Description
Details	1	98	submission	MOSPD1	motile sperm domain containing 1
Details	2	97	submission	CDK13	cyclin dependent kinase 13
Details	3	97	submission	VAPA	VAMP associated protein A
Details	4	97	submission	NEMF	nuclear export mediator factor
Details	5	96	submission	LYPD6	LY6/PLAUR domain containing 6
Details	6	96	submission	P2RY13	purinergic receptor P2Y13
Details	7	96	submission	KIAA1324L	KIAA1324 like
Details	8	95	submission	WDR7	WD repeat domain 7
Details	9	94	submission	INPP5E	inositol polyphosphate-5-phosphatase E
Details	10	94	submission	STAG2	stromal antigen 2
Details	11	94	submission	PHF6	PHD finger protein 6
Details	12	94	submission	RWDD4	RWD domain containing 4
Details	13	93	submission	MAP3K2	mitogen-activated protein kinase kinase kinase 2
Details	14	93	submission	HMCN1	hemicentin 1
Details	15	92	submission	SMURF2	SMAD specific E3 ubiquitin protein ligase 2
Details	16	92	submission	DISC1	DISC1 scaffold protein
Details	17	91	submission	MLLT3	MLLT3, super elongation complex subunit
Details	18	91	submission	ZRANB1	zinc finger RANBP2-type containing 1
Details	19	91	submission	MS4A7	membrane spanning 4-domains A7
Details	20	91	submission	CGRRF1	cell growth regulator with ring finger domain 1
Details	21	91	submission	LIN28B	lin-28 homolog B
Details	22	91	submission	PPP2R5E	protein phosphatase 2 regulatory subunit B'epsilon
Details	23	91	submission	WWC2	WW and C2 domain containing 2
Details	24	90	submission	MTM1	myotubularin 1
Details	25	90	submission	ADD2	adducin 2
Details	26	90	submission	TTK	TTK protein kinase
Details	27	90	submission	GPR18	G protein-coupled receptor 18

Details	28	90	submission	UNC5D	unc-5 netrin receptor D
Details	29	90	submission	LACTB	lactamase beta
Details	30	90	submission	COL5A2	collagen type V alpha 2 chain
Details	31	90	submission	IL6ST	interleukin 6 signal transducer
Details	32	90	submission	FLT1	fms related tyrosine kinase 1
Details	33	89	submission	PKD2	polycystin 2, transient receptor potential cation channel
Details	34	89	submission	EBF2	EBF transcription factor 2
Details	35	89	submission	KLHL15	kelch like family member 15
Details	36	89	submission	PTCD2	pentatricopeptide repeat domain 2
Details	37	89	submission	GRIA2	glutamate ionotropic receptor AMPA type subunit 2
Details	38	89	submission	DDAH1	dimethylarginine dimethylaminohydrolase 1
Details	39	89	submission	SEC63	SEC63 homolog, protein translocation regulator
Details	40	89	submission	RTL9	retrotransposon Gag like 9
Details	41	89	submission	SREK1IP1	SREK1 interacting protein 1
Details	42	89	submission	NABP1	nucleic acid binding protein 1
Details	43	89	submission	KDELR1	KDEL endoplasmic reticulum protein retention receptor 1
Details	44	88	submission	HPCAL4	hippocalcin like 4
Details	45	88	submission	CXCL9	C-X-C motif chemokine ligand 9
Details	46	88	submission	FGA	fibrinogen alpha chain
Details	47	88	submission	DENND6A	DENN domain containing 6A
Details	48	88	submission	KAT6A	lysine acetyltransferase 6A
Details	49	88	submission	IKZF3	IKAROS family zinc finger 3
Details	50	88	submission	SYNRG	synergin gamma
Details	51	88	submission	NDUFS1	NADH:ubiquinone oxidoreductase core subunit S1
Details	52	88	submission	PGF	placental growth factor
Details	53	88	submission	DCLK1	doublecortin like kinase 1
Details	54	88	submission	PCMTD1	protein-L-isoaspartate (D-aspartate) O-methyltransferase domain containing 1
Details	55	88	submission	IL7R	interleukin 7 receptor
Details	56	87	submission	ZNF714	zinc finger protein 714
Details	57	87	submission	UBE2V2	ubiquitin conjugating enzyme E2 V2
Details	58	87	submission	TEAD1	TEA domain transcription factor 1
Details	59	87	submission	WDR26	WD repeat domain 26
Details	60	87	submission	LETM2	leucine zipper and EF-hand containing transmembrane protein 2
Details	61	87	submission	USF3	upstream transcription factor family member 3
Details	62	87	submission	PHTF2	putative homeodomain transcription factor 2

Details	63	87	submission	STAG1	stromal antigen 1
Details	64	87	submission	ACTR2	ARP2 actin related protein 2 homolog
Details	65	87	submission	NLN	neurolysin
Details	66	87	submission	EPHA4	EPH receptor A4
Details	67	86	submission	TRIM32	tripartite motif containing 32
Details	68	86	submission	PTP4A1	protein tyrosine phosphatase type IVA, member 1
Details	69	86	submission	ZNF610	zinc finger protein 610
Details	70	86	submission	DCTN4	dynactin subunit 4
Details	71	86	submission	RPGR	retinitis pigmentosa GTPase regulator
Details	72	86	submission	SECISBP2L	SECIS binding protein 2 like
Details	73	86	submission	NPC2	NPC intracellular cholesterol transporter 2
Details	74	86	submission	C1D	C1D nuclear receptor corepressor
Details	75	86	submission	TRDN	triadin
Details	76	86	submission	GABRB2	gamma-aminobutyric acid type A receptor beta2 subunit
Details	77	86	submission	PLAGL1	PLAG1 like zinc finger 1
Details	78	86	submission	CHRM2	cholinergic receptor muscarinic 2
Details	79	86	submission	ZKSCAN7	zinc finger with KRAB and SCAN domains 7
Details	80	86	submission	ENTPD1	ectonucleoside triphosphate diphosphohydrolase 1
Details	81	85	submission	MTMR9	myotubularin related protein 9
Details	82	85	submission	MED21	mediator complex subunit 21
Details	83	85	submission	TMEM65	transmembrane protein 65
Details	84	85	submission	MS4A1	membrane spanning 4-domains A1
Details	85	85	submission	PHLPP1	PH domain and leucine rich repeat protein phosphatase 1
Details	86	85	submission	NT5C3A	5'-nucleotidase, cytosolic IIIA
Details	87	85	submission	FAM78A	family with sequence similarity 78 member A
Details	88	85	submission	EMB	embigin
Details	89	85	submission	SELENOF	selenoprotein F
Details	90	85	submission	TRAPPC11	trafficking protein particle complex 11
Details	91	85	submission	H1F0	H1 histone family member 0
Details	92	85	submission	NXPH2	neurexophilin 2
Details	93	85	submission	PCGF5	polycomb group ring finger 5
Details	94	85	submission	MDM4	MDM4, p53 regulator
Details	95	84	submission	SRSF10	serine and arginine rich splicing factor 10
Details	96	84	submission	RSRC2	arginine and serine rich coiled-coil 2
Details	97	84	submission	CMTM6	CKLF like MARVEL transmembrane domain containing 6
Details	98	84	submission	TMTC4	transmembrane and tetratricopeptide repeat containing 4

Details	99	84	submission	NCKAP5	NCK associated protein 5
Details	100	84	submission	SYDE2	synapse defective Rho GTPase homolog 2
Details	101	84	submission	YTHDF1	YTH N6-methyladenosine RNA binding protein 1
Details	102	84	submission	GAB2	GRB2 associated binding protein 2
Details	103	84	submission	PRKAA2	protein kinase AMP-activated catalytic subunit alpha 2
Details	104	84	submission	KMT2C	lysine methyltransferase 2C
Details	105	84	submission	ITGB8	integrin subunit beta 8
Details	106	84	submission	CEP350	centrosomal protein 350
Details	107	83	submission	ARNT2	aryl hydrocarbon receptor nuclear translocator 2
Details	108	83	submission	MPP5	membrane palmitoylated protein 5
Details	109	83	submission	ZIC3	Zic family member 3
Details	110	83	submission	FMN1	formin 1
Details	111	83	submission	TMEM59	transmembrane protein 59
Details	112	83	submission	FRS2	fibroblast growth factor receptor substrate 2
Details	113	83	submission	SLC25A13	solute carrier family 25 member 13
Details	114	83	submission	DCX	doublecortin
Details	115	83	submission	SETDB2	SET domain bifurcated histone lysine methyltransferase 2
Details	116	82	submission	HTRA3	HtrA serine peptidase 3
Details	117	82	submission	ZBTB8B	zinc finger and BTB domain containing 8B
Details	118	82	submission	MTRF1L	mitochondrial translational release factor 1 like
Details	119	82	submission	TMC8	transmembrane channel like 8
Details	120	82	submission	POU2F3	POU class 2 homeobox 3
Details	121	82	submission	SEMA3D	semaphorin 3D
Details	122	82	submission	USP8	ubiquitin specific peptidase 8
Details	123	82	submission	UBE2Q2	ubiquitin conjugating enzyme E2 Q2
Details	124	82	submission	SGCZ	sarcoglycan zeta
Details	125	82	submission	PRKD1	protein kinase D1
Details	126	82	submission	ZNF780B	zinc finger protein 780B
Details	127	82	submission	PANK1	pantothenate kinase 1
Details	128	82	submission	TP53TG3D	TP53 target 3D
Details	129	82	submission	RCBTB1	RCC1 and BTB domain containing protein 1
Details	130	82	submission	KPRP	keratinocyte proline rich protein
Details	131	81	submission	ADM2	adrenomedullin 2
Details	132	81	submission	GPA33	glycoprotein A33
Details	133	81	submission	AGFG1	ArfGAP with FG repeats 1
Details	134	81	submission	CALU	calumenin

Details	135	81	submission	TMPPE	transmembrane protein with metallophosphoesterase domain
Details	136	81	submission	NRCAM	neuronal cell adhesion molecule
Details	137	81	submission	ENPEP	glutamyl aminopeptidase
Details	138	81	submission	SNIP1	Smad nuclear interacting protein 1
Details	139	81	submission	CHFR	checkpoint with forkhead and ring finger domains
Details	140	81	submission	KPNA1	karyopherin subunit alpha 1
Details	141	81	submission	PPP1CA	protein phosphatase 1 catalytic subunit alpha
Details	142	81	submission	CSRNP3	cysteine and serine rich nuclear protein 3
Details	143	81	submission	GPM6A	glycoprotein M6A
Details	144	80	submission	ZNF616	zinc finger protein 616
Details	145	80	submission	ZNF577	zinc finger protein 577
Details	146	80	submission	ZBTB10	zinc finger and BTB domain containing 10
Details	147	80	submission	TIMM10B	translocase of inner mitochondrial membrane 10B
Details	148	80	submission	SYNCRIP	synaptotagmin binding cytoplasmic RNA interacting protein
Details	149	80	submission	GABRA4	gamma-aminobutyric acid type A receptor alpha4 subunit
Details	150	80	submission	MAP6	microtubule associated protein 6
Details	151	80	submission	CCNT2	cyclin T2
Details	152	80	submission	ZBTB20	zinc finger and BTB domain containing 20
Details	153	80	submission	NTRK2	neurotrophic receptor tyrosine kinase 2
Details	154	80	submission	HIST2H2BF	histone cluster 2 H2B family member f
Details	155	80	submission	MZT1	mitotic spindle organizing protein 1
Details	156	80	submission	PPP1R3A	protein phosphatase 1 regulatory subunit 3A
Details	157	80	submission	TSPAN2	tetraspanin 2
Details	158	79	submission	TNPO1	transportin 1
Details	159	79	submission	TBC1D12	TBC1 domain family member 12
Details	160	79	submission	MTDH	metadherin
Details	161	79	submission	PTPRB	protein tyrosine phosphatase, receptor type B
Details	162	79	submission	ZC2HC1C	zinc finger C2HC-type containing 1C
Details	163	79	submission	LCORL	ligand dependent nuclear receptor corepressor like
Details	164	79	submission	CELF4	CUGBP Elav-like family member 4
Details	165	79	submission	ZMYM4	zinc finger MYM-type containing 4
Details	166	79	submission	HIC2	HIC ZBTB transcriptional repressor 2
Details	167	79	submission	VPS50	VPS50, EARP/GARPII complex subunit
Details	168	79	submission	FBXW2	F-box and WD repeat domain containing 2
Details	169	78	submission	DGAT2	diacylglycerol O-acyltransferase 2

Details	170	78	submission	STX17	syntaxin 17
Details	171	78	submission	ATP5MG	ATP synthase membrane subunit g
Details	172	78	submission	BNIP2	BCL2 interacting protein 2
Details	173	78	submission	SPINK6	serine peptidase inhibitor, Kazal type 6
Details	174	78	submission	TRIM66	tripartite motif containing 66
Details	175	78	submission	COL6A3	collagen type VI alpha 3 chain
Details	176	78	submission	AP3B1	adaptor related protein complex 3 subunit beta 1
Details	177	78	submission	MCOLN3	mucolipin 3
Details	178	78	submission	CPEB2	cytoplasmic polyadenylation element binding protein 2
Details	179	78	submission	NAA25	N(alpha)-acetyltransferase 25, NatB auxiliary subunit
Details	180	78	submission	ASCC3	activating signal cointegrator 1 complex subunit 3
Details	181	78	submission	ST8SIA4	ST8 alpha-N-acetyl-neuraminide alpha-2,8-sialyltransferase 4
Details	182	78	submission	PRKACA	protein kinase cAMP-activated catalytic subunit alpha
Details	183	78	submission	MICU3	mitochondrial calcium uptake family member 3
Details	184	77	submission	MSH3	mutS homolog 3
Details	185	77	submission	WBP1L	WW domain binding protein 1 like
Details	186	77	submission	WDR20	WD repeat domain 20
Details	187	77	submission	RHOU	ras homolog family member U
Details	188	77	submission	RNF141	ring finger protein 141
Details	189	77	submission	PDP1	pyruvate dehyrogenase phosphatase catalytic subunit 1
Details	190	77	submission	CDK7	cyclin dependent kinase 7
Details	191	77	submission	ZNF583	zinc finger protein 583
Details	192	76	submission	DOCK4	dedicator of cytokinesis 4
Details	193	76	submission	SOX6	SRY-box 6
Details	194	76	submission	SRR	serine racemase
Details	195	76	submission	EFR3A	EFR3 homolog A
Details	196	76	submission	PQLC2L	PQ loop repeat containing 2 like
Details	197	76	submission	HOXA5	homeobox A5
Details	198	76	submission	C1QTNF7	C1q and TNF related 7
Details	199	76	submission	ACKR3	atypical chemokine receptor 3
Details	200	76	submission	KIF3A	kinesin family member 3A
Details	201	76	submission	USP24	ubiquitin specific peptidase 24
Details	202	76	submission	LY6G5B	lymphocyte antigen 6 family member G5B
Details	203	75	submission	CPEB4	cytoplasmic polyadenylation element binding protein 4
Details	204	75	submission	MACO1	macoilin 1

Details	205	75	submission	SNAP91	synaptosome associated protein 91
Details	206	75	submission	RBFOX2	RNA binding fox-1 homolog 2
Details	207	75	submission	HGF	hepatocyte growth factor
Details	208	75	submission	GJC1	gap junction protein gamma 1
Details	209	75	submission	CACNA1C	calcium voltage-gated channel subunit alpha1 C
Details	210	75	submission	TMEM33	transmembrane protein 33
Details	211	75	submission	MOGAT2	monoacylglycerol O-acyltransferase 2
Details	212	75	submission	ANKRD28	ankyrin repeat domain 28
Details	213	75	submission	TMEM135	transmembrane protein 135
Details	214	75	submission	DNM3	dynamin 3
Details	215	75	submission	PLD5	phospholipase D family member 5
Details	216	75	submission	ARFGEF3	ARFGEF family member 3
Details	217	75	submission	PLAGL2	PLAG1 like zinc finger 2
Details	218	75	submission	DERL1	derlin 1
Details	219	75	submission	KIAA0825	KIAA0825
Details	220	74	submission	VASH2	vasohibin 2
Details	221	74	submission	TMEM178B	transmembrane protein 178B
Details	222	74	submission	TGFB2	transforming growth factor beta 2
Details	223	74	submission	PMEPA1	prostate transmembrane protein, androgen induced 1
Details	224	74	submission	CDH13	cadherin 13
Details	225	74	submission	TNFAIP1	TNF alpha induced protein 1
Details	226	74	submission	CHST6	carbohydrate sulfotransferase 6
Details	227	74	submission	PEX11B	peroxisomal biogenesis factor 11 beta
Details	228	74	submission	CCDC88A	coiled-coil domain containing 88A
Details	229	74	submission	FOXJ3	forkhead box J3
Details	230	74	submission	FAR1	fatty acyl-CoA reductase 1
Details	231	74	submission	DGKB	diacylglycerol kinase beta
Details	232	74	submission	ZNF264	zinc finger protein 264
Details	233	74	submission	CCDC68	coiled-coil domain containing 68
Details	234	73	submission	ZNF845	zinc finger protein 845
Details	235	73	submission	RUFY2	RUN and FYVE domain containing 2
Details	236	73	submission	TRAF5	TNF receptor associated factor 5
Details	237	73	submission	GOLPH3L	golgi phosphoprotein 3 like
Details	238	73	submission	CCNC	cyclin C
Details	239	73	submission	HCFC2	host cell factor C2
Details	240	73	submission	TCEANC2	transcription elongation factor A N-terminal and central domain containing 2
Details	241	73	submission	FAM124B	family with sequence similarity 124 member B

Details	242	73	submission	LALBA	lactalbumin alpha
Details	243	73	submission	CYCS	cytochrome c, somatic
Details	244	73	submission	CDC26	cell division cycle 26
Details	245	73	submission	ZNF561	zinc finger protein 561
Details	246	73	submission	RPL22L1	ribosomal protein L22 like 1
Details	247	72	submission	TRIM5	tripartite motif containing 5
Details	248	72	submission	NMT2	N-myristoyltransferase 2
Details	249	72	submission	AP5M1	adaptor related protein complex 5 subunit mu 1
Details	250	72	submission	LARP4B	La ribonucleoprotein domain family member 4B
Details	251	72	submission	DPP10	dipeptidyl peptidase like 10
Details	252	72	submission	PNISR	PNN interacting serine and arginine rich protein
Details	253	72	submission	CTNNA3	catenin alpha 3
Details	254	71	submission	LGALSL	galectin like
Details	255	71	submission	AICDA	activation induced cytidine deaminase
Details	256	71	submission	FPR2	formyl peptide receptor 2
Details	257	71	submission	TMEM213	transmembrane protein 213
Details	258	71	submission	SPTBN1	spectrin beta, non-erythrocytic 1
Details	259	71	submission	USP15	ubiquitin specific peptidase 15
Details	260	71	submission	CD86	CD86 molecule
Details	261	71	submission	SLC26A9	solute carrier family 26 member 9
Details	262	71	submission	CSTF1	cleavage stimulation factor subunit 1
Details	263	71	submission	ETNK1	ethanolamine kinase 1
Details	264	71	submission	GIT2	GIT ArfGAP 2
Details	265	71	submission	BICC1	BicC family RNA binding protein 1
Details	266	71	submission	GOLT1B	golgi transport 1B
Details	267	71	submission	JUP	junction plakoglobin
Details	268	71	submission	SLC25A23	solute carrier family 25 member 23
Details	269	70	submission	EPHA8	EPH receptor A8
Details	270	70	submission	SCAPER	S-phase cyclin A associated protein in the ER
Details	271	70	submission	SRSF3	serine and arginine rich splicing factor 3
Details	272	70	submission	ASPH	aspartate beta-hydroxylase
Details	273	70	submission	KLF9	Kruppel like factor 9
Details	274	70	submission	OSBP	oxysterol binding protein
Details	275	70	submission	GALNT3	polypeptide N-acetylgalactosaminyltransferase 3
Details	276	70	submission	CEP97	centrosomal protein 97
Details	277	70	submission	RAP1A	RAP1A, member of RAS oncogene family

Details	278	70	submission	GGCT	gamma-glutamylcyclotransferase
Details	279	70	submission	SH2B3	SH2B adaptor protein 3
Details	280	70	submission	DLG1	discs large MAGUK scaffold protein 1
Details	281	70	submission	METTL8	methyltransferase like 8
Details	282	69	submission	ZNHIT6	zinc finger HIT-type containing 6
Details	283	69	submission	AAK1	AP2 associated kinase 1
Details	284	69	submission	TMEM41A	transmembrane protein 41A
Details	285	69	submission	EPC1	enhancer of polycomb homolog 1
Details	286	69	submission	TRIM2	tripartite motif containing 2
Details	287	69	submission	EIF2AK3	eukaryotic translation initiation factor 2 alpha kinase 3
Details	288	69	submission	CENPK	centromere protein K
Details	289	69	submission	CNOT7	CCR4-NOT transcription complex subunit 7
Details	290	69	submission	KRI1	KRI1 homolog
Details	291	69	submission	ZNF148	zinc finger protein 148
Details	292	68	submission	TP53INP1	tumor protein p53 inducible nuclear protein 1
Details	293	68	submission	BCAT1	branched chain amino acid transaminase 1
Details	294	68	submission	VAT1L	vesicle amine transport 1 like
Details	295	68	submission	ZDHHC3	zinc finger DHHC-type containing 3
Details	296	68	submission	TMTC1	transmembrane and tetratricopeptide repeat containing 1
Details	297	68	submission	RBMS3	RNA binding motif single stranded interacting protein 3
Details	298	68	submission	COPG1	coatomer protein complex subunit gamma 1
Details	299	68	submission	ZMAT3	zinc finger matrin-type 3
Details	300	68	submission	SRSF4	serine and arginine rich splicing factor 4
Details	301	68	submission	NKTR	natural killer cell triggering receptor
Details	302	68	submission	OGFOD1	2-oxoglutarate and iron dependent oxygenase domain containing 1
Details	303	68	submission	ACVR1C	activin A receptor type 1C
Details	304	68	submission	ZEB2	zinc finger E-box binding homeobox 2
Details	305	68	submission	ALPG	alkaline phosphatase, germ cell
Details	306	68	submission	CDH11	cadherin 11
Details	307	68	submission	GBP6	guanylate binding protein family member 6
Details	308	68	submission	EEF2K	eukaryotic elongation factor 2 kinase
Details	309	68	submission	SEPT7	septin 7
Details	310	68	submission	PTPRK	protein tyrosine phosphatase, receptor type K
Details	311	68	submission	AJAP1	adherens junctions associated protein 1
Details	312	68	submission	PURB	purine rich element binding protein B
Details	313	67	submission	LSM11	LSM11, U7 small nuclear RNA associated

Details	314	67	submission	ATG2B	autophagy related 2B
Details	315	67	submission	SMAD2	SMAD family member 2
Details	316	67	submission	CNOT6	CCR4-NOT transcription complex subunit 6
Details	317	67	submission	TPP1	tripeptidyl peptidase 1
Details	318	67	submission	SVEP1	sushi, von Willebrand factor type A, EGF and pentraxin domain containing 1
Details	319	67	submission	CA12	carbonic anhydrase 12
Details	320	67	submission	QKI	QKI, KH domain containing RNA binding
Details	321	67	submission	MYBL1	MYB proto-oncogene like 1
Details	322	67	submission	B3GNT2	UDP-GlcNAc:betaGal beta-1,3-N-acetylglucosaminyltransferase 2
Details	323	67	submission	TNRC6B	trinucleotide repeat containing 6B
Details	324	67	submission	ODC1	ornithine decarboxylase 1
Details	325	66	submission	ADAMDEC1	ADAM like decysin 1
Details	326	66	submission	ARIH2	ariadne RBR E3 ubiquitin protein ligase 2
Details	327	66	submission	GPX5	glutathione peroxidase 5
Details	328	66	submission	EIF4G3	eukaryotic translation initiation factor 4 gamma 3
Details	329	66	submission	KLHL2	kelch like family member 2
Details	330	66	submission	EDDM3B	epididymal protein 3B
Details	331	66	submission	GUCY1A2	guanylate cyclase 1 soluble subunit alpha 2
Details	332	66	submission	TTC33	tetratricopeptide repeat domain 33
Details	333	66	submission	MBNL2	muscleblind like splicing regulator 2
Details	334	66	submission	CEACAM5	carcinoembryonic antigen related cell adhesion molecule 5
Details	335	66	submission	GZF1	GDNF inducible zinc finger protein 1
Details	336	65	submission	SPRY3	sprouty RTK signaling antagonist 3
Details	337	65	submission	ALOX12	arachidonate 12-lipoxygenase, 12S type
Details	338	65	submission	NES	nestin
Details	339	65	submission	YPEL2	yippee like 2
Details	340	65	submission	GABRG2	gamma-aminobutyric acid type A receptor gamma2 subunit
Details	341	65	submission	CHCHD1	coiled-coil-helix-coiled-coil-helix domain containing 1
Details	342	65	submission	RALA	RAS like proto-oncogene A
Details	343	65	submission	GDE1	glycerophosphodiester phosphodiesterase 1
Details	344	65	submission	AIG1	androgen induced 1
Details	345	65	submission	NHS	NHS actin remodeling regulator
Details	346	65	submission	MFAP3L	microfibril associated protein 3 like
Details	347	65	submission	STXBP1	syntaxin binding protein 1
Details	348	65	submission	JMY	junction mediating and regulatory protein, p53 cofactor
Details	349	65	submission	ZMPSTE24	zinc metallopeptidase STE24

Details	350	65	submission	RBSN	rabenosyn, RAB effector
Details	351	65	submission	FAM120C	family with sequence similarity 120C
Details	352	65	submission	ZNF521	zinc finger protein 521
Details	353	65	submission	CDADC1	cytidine and dCMP deaminase domain containing 1
Details	354	65	submission	TBC1D30	TBC1 domain family member 30
Details	355	64	submission	MAP9	microtubule associated protein 9
Details	356	64	submission	EPHX3	epoxide hydrolase 3
Details	357	64	submission	USP13	ubiquitin specific peptidase 13
Details	358	64	submission	NDST3	N-deacetylase and N-sulfotransferase 3
Details	359	64	submission	PODNL1	podocan like 1
Details	360	64	submission	LMOD1	leiomodin 1
Details	361	64	submission	RNF145	ring finger protein 145
Details	362	64	submission	RAD21	RAD21 cohesin complex component
Details	363	64	submission	UBE2W	ubiquitin conjugating enzyme E2 W
Details	364	64	submission	GTF2E2	general transcription factor IIE subunit 2
Details	365	64	submission	KCTD14	potassium channel tetramerization domain containing 14
Details	366	64	submission	AMPD3	adenosine monophosphate deaminase 3
Details	367	64	submission	ANO1	anoctamin 1
Details	368	64	submission	NPR3	natriuretic peptide receptor 3
Details	369	64	submission	KAZALD1	Kazal type serine peptidase inhibitor domain 1
Details	370	64	submission	ZNF608	zinc finger protein 608
Details	371	64	submission	EMC1	ER membrane protein complex subunit 1
Details	372	63	submission	CPB1	carboxypeptidase B1
Details	373	63	submission	ADAT2	adenosine deaminase, tRNA specific 2
Details	374	63	submission	SPOCK1	SPARC (osteonectin), cwcv and kazal like domains proteoglycan 1
Details	375	63	submission	KSR2	kinase suppressor of ras 2
Details	376	63	submission	ADIPOQ	adiponectin, C1Q and collagen domain containing
Details	377	63	submission	WDR5B	WD repeat domain 5B
Details	378	63	submission	SLC37A3	solute carrier family 37 member 3
Details	379	63	submission	ZNF813	zinc finger protein 813
Details	380	63	submission	PFAS	phosphoribosylformylglycinamidine synthase
Details	381	63	submission	DIDO1	death inducer-obliterator 1
Details	382	63	submission	EIF3H	eukaryotic translation initiation factor 3 subunit H
Details	383	63	submission	ONECUT2	one cut homeobox 2
Details	384	62	submission	POLR2F	RNA polymerase II subunit F
Details	385	62	submission	SLC35A5	solute carrier family 35 member A5

Details	386	62	submission	CLMN	calmin
Details	387	62	submission	YAP1	Yes associated protein 1
Details	388	62	submission	WDR36	WD repeat domain 36
Details	389	62	submission	LIPG	lipase G, endothelial type
Details	390	62	submission	TMEM155	transmembrane protein 155
Details	391	62	submission	SCAI	suppressor of cancer cell invasion
Details	392	62	submission	EIF5A2	eukaryotic translation initiation factor 5A2
Details	393	62	submission	ITFG1	integrin alpha FG-GAP repeat containing 1
Details	394	62	submission	MSMO1	methylsterol monooxygenase 1
Details	395	62	submission	WFDC13	WAP four-disulfide core domain 13
Details	396	62	submission	ACPP	acid phosphatase, prostate
Details	397	62	submission	RAB39A	RAB39A, member RAS oncogene family
Details	398	62	submission	ELFN2	extracellular leucine rich repeat and fibronectin type III domain containing 2
Details	399	62	submission	REEP1	receptor accessory protein 1
Details	400	62	submission	PRELID2	PRELI domain containing 2
Details	401	62	submission	MACC1	MET transcriptional regulator MACC1
Details	402	62	submission	CTDSP2	CTD small phosphatase 2
Details	403	62	submission	PRPF4B	pre-mRNA processing factor 4B
Details	404	62	submission	USP46	ubiquitin specific peptidase 46
Details	405	61	submission	PDLIM1	PDZ and LIM domain 1
Details	406	61	submission	USP12	ubiquitin specific peptidase 12
Details	407	61	submission	FAM71F2	family with sequence similarity 71 member F2
Details	408	61	submission	GNAQ	G protein subunit alpha q
Details	409	61	submission	DDX3X	DEAD-box helicase 3 X-linked
Details	410	61	submission	TTC14	tetratricopeptide repeat domain 14
Details	411	61	submission	RABGAP1L	RAB GTPase activating protein 1 like
Details	412	61	submission	TUBB1	tubulin beta 1 class VI
Details	413	61	submission	GNG12	G protein subunit gamma 12
Details	414	61	submission	CEP41	centrosomal protein 41
Details	415	61	submission	PCDH10	protocadherin 10
Details	416	61	submission	C1GALT1	core 1 synthase, glycoprotein-N-acetylgalactosamine 3-beta-galactosyltransferase 1
Details	417	61	submission	PEX12	peroxisomal biogenesis factor 12
Details	418	61	submission	ARGLU1	arginine and glutamate rich 1
Details	419	61	submission	MARCH1	membrane associated ring-CH-type finger 1
Details	420	60	submission	ZNF677	zinc finger protein 677
Details	421	60	submission	SEPT10	septin 10

Details	422	60	submission	GNA12	G protein subunit alpha 12
Details	423	60	submission	PARP9	poly(ADP-ribose) polymerase family member 9
Details	424	60	submission	NUMA1	nuclear mitotic apparatus protein 1
Details	425	60	submission	BET1	Bet1 golgi vesicular membrane trafficking protein
Details	426	60	submission	YKT6	YKT6 v-SNARE homolog
Details	427	60	submission	TMBIM6	transmembrane BAX inhibitor motif containing 6
Details	428	60	submission	ZNF451	zinc finger protein 451
Details	429	60	submission	PTPN11	protein tyrosine phosphatase, non-receptor type 11
Details	430	60	submission	ACAP3	ArfGAP with coiled-coil, ankyrin repeat and PH domains 3
Details	431	60	submission	CLP1	cleavage and polyadenylation factor I subunit 1
Details	432	60	submission	GNL3L	G protein nucleolar 3 like
Details	433	60	submission	WEE2	WEE1 homolog 2
Details	434	60	submission	ST18	ST18, C2H2C-type zinc finger
Details	435	60	submission	RAD51B	RAD51 paralog B
Details	436	59	submission	HPS4	HPS4, biogenesis of lysosomal organelles complex 3 subunit 2
Details	437	59	submission	GAREM1	GRB2 associated regulator of MAPK1 subtype 1
Details	438	59	submission	CSPG5	chondroitin sulfate proteoglycan 5
Details	439	59	submission	PCDHB7	protocadherin beta 7
Details	440	59	submission	KCTD7	potassium channel tetramerization domain containing 7
Details	441	59	submission	NDUFC2-KCTD14	NDUFC2-KCTD14 readthrough
Details	442	59	submission	MTX3	metaxin 3
Details	443	59	submission	KRT24	keratin 24
Details	444	59	submission	BCL2	BCL2, apoptosis regulator
Details	445	59	submission	NIPA1	NIPA magnesium transporter 1
Details	446	59	submission	CIAO2B	cytosolic iron-sulfur assembly component 2B
Details	447	59	submission	SNTB2	syntrophin beta 2
Details	448	59	submission	IPO7	importin 7
Details	449	59	submission	NFIB	nuclear factor I B
Details	450	59	submission	LCOR	ligand dependent nuclear receptor corepressor
Details	451	59	submission	ISY1	ISY1 splicing factor homolog
Details	452	59	submission	RNF32	ring finger protein 32
Details	453	59	submission	RBCK1	RANBP2-type and C3HC4-type zinc finger containing 1
Details	454	59	submission	RBM22	RNA binding motif protein 22

Details	455	59	submission	RSPRY1	ring finger and SPRY domain containing 1
Details	456	59	submission	TRPM7	transient receptor potential cation channel subfamily M member 7
Details	457	59	submission	SYT7	synaptotagmin 7
Details	458	59	submission	COX15	cytochrome c oxidase assembly homolog COX15
Details	459	59	submission	LIFR	LIF receptor alpha
Details	460	59	submission	ZNF491	zinc finger protein 491
Details	461	59	submission	ASTN1	astrotactin 1
Details	462	58	submission	ETV3	ETS variant 3
Details	463	58	submission	ARSD	arylsulfatase D
Details	464	58	submission	MYO5A	myosin VA
Details	465	58	submission	BNC2	basonuclin 2
Details	466	58	submission	EIF4G1	eukaryotic translation initiation factor 4 gamma 1
Details	467	58	submission	IRAK3	interleukin 1 receptor associated kinase 3
Details	468	58	submission	CASP7	caspase 7
Details	469	58	submission	CDC5L	cell division cycle 5 like
Details	470	58	submission	MRPS36	mitochondrial ribosomal protein S36
Details	471	58	submission	LRG1	leucine rich alpha-2-glycoprotein 1
Details	472	58	submission	TSPAN17	tetraspanin 17
Details	473	58	submission	CREBRF	CREB3 regulatory factor
Details	474	58	submission	C12orf73	chromosome 12 open reading frame 73
Details	475	58	submission	TXNDC5	thioredoxin domain containing 5
Details	476	58	submission	TUBE1	tubulin epsilon 1
Details	477	57	submission	TMF1	TATA element modulatory factor 1
Details	478	57	submission	CDC42SE2	CDC42 small effector 2
Details	479	57	submission	SIRPG	signal regulatory protein gamma
Details	480	57	submission	SCARB1	scavenger receptor class B member 1
Details	481	57	submission	AFF3	AF4/FMR2 family member 3
Details	482	57	submission	VPS13D	vacuolar protein sorting 13 homolog D
Details	483	57	submission	ZNF470	zinc finger protein 470
Details	484	57	submission	CNOT4	CCR4-NOT transcription complex subunit 4
Details	485	57	submission	ZNF154	zinc finger protein 154
Details	486	57	submission	JAG1	jagged 1
Details	487	57	submission	SERPINB9	serpin family B member 9
Details	488	57	submission	TCFL5	transcription factor like 5
Details	489	57	submission	C9orf47	chromosome 9 open reading frame 47
Details	490	57	submission	TEX261	testis expressed 261
Details	491	57	submission	PARD6B	par-6 family cell polarity regulator beta

Details	492	57	submission	CRYZL1	crystallin zeta like 1
Details	493	57	submission	KLHL8	kelch like family member 8
Details	494	57	submission	TIFA	TRAF interacting protein with forkhead associated domain
Details	495	57	submission	RPL22	ribosomal protein L22
Details	496	57	submission	MRI1	methylthioribose-1-phosphate isomerase 1
Details	497	57	submission	FBXW8	F-box and WD repeat domain containing 8
Details	498	57	submission	TMEM173	transmembrane protein 173
Details	499	57	submission	ACYP2	acylphosphatase 2
Details	500	57	submission	KDELR3	KDEL endoplasmic reticulum protein retention receptor 3
Details	501	57	submission	KCNJ13	potassium voltage-gated channel subfamily J member 13
Details	502	57	submission	KPNA6	karyopherin subunit alpha 6
Details	503	57	submission	APTX	aprataxin
Details	504	57	submission	CRNKL1	crooked neck pre-mRNA splicing factor 1
Details	505	56	submission	DCDC2	doublecortin domain containing 2
Details	506	56	submission	GSK3B	glycogen synthase kinase 3 beta
Details	507	56	submission	NDNF	neuron derived neurotrophic factor
Details	508	56	submission	GAR1	GAR1 ribonucleoprotein
Details	509	56	submission	MRPS11	mitochondrial ribosomal protein S11
Details	510	56	submission	ZBTB6	zinc finger and BTB domain containing 6
Details	511	56	submission	TKTL2	transketolase like 2
Details	512	56	submission	GCLM	glutamate-cysteine ligase modifier subunit
Details	513	56	submission	ZNF662	zinc finger protein 662
Details	514	56	submission	ATP1B1	ATPase Na+/K+ transporting subunit beta 1
Details	515	56	submission	HBP1	HMG-box transcription factor 1
Details	516	56	submission	PTAR1	protein prenyltransferase alpha subunit repeat containing 1
Details	517	56	submission	SOCS5	suppressor of cytokine signaling 5
Details	518	56	submission	ARHGAP33	Rho GTPase activating protein 33
Details	519	55	submission	PRPF18	pre-mRNA processing factor 18
Details	520	55	submission	NAA30	N(alpha)-acetyltransferase 30, NatC catalytic subunit
Details	521	55	submission	CCT6A	chaperonin containing TCP1 subunit 6A
Details	522	55	submission	KRAS	KRAS proto-oncogene, GTPase
Details	523	55	submission	TRIM49D1	tripartite motif containing 49D1
Details	524	55	submission	GPD1L	glycerol-3-phosphate dehydrogenase 1 like
Details	525	55	submission	B4GAT1	beta-1,4-glucuronyltransferase 1
Details	526	55	submission	GRIK2	glutamate ionotropic receptor kainate type subunit 2
Details	527	55	submission	SPRY4	sprouty RTK signaling antagonist 4

Details	528	55	submission	PDK3	pyruvate dehydrogenase kinase 3
Details	529	55	submission	SLC1A2	solute carrier family 1 member 2
Details	530	55	submission	SLC16A7	solute carrier family 16 member 7
Details	531	55	submission	N4BP1	NEDD4 binding protein 1
Details	532	55	submission	DENND4C	DENN domain containing 4C
Details	533	55	submission	KCNMB2	potassium calcium-activated channel subfamily M regulatory beta subunit 2
Details	534	55	submission	ZNF790	zinc finger protein 790
Details	535	55	submission	NFKBIE	NFKB inhibitor epsilon
Details	536	55	submission	IKZF2	IKAROS family zinc finger 2
Details	537	55	submission	GOLPH3	golgi phosphoprotein 3
Details	538	55	submission	MGAT5B	alpha-1,6-mannosylglycoprotein 6-beta-N-acetylglucosaminyltransferase B
Details	539	55	submission	CWC27	CWC27 spliceosome associated protein homolog
Details	540	55	submission	GALNT13	polypeptide N-acetylgalactosaminyltransferase 13
Details	541	55	submission	TNFAIP8L1	TNF alpha induced protein 8 like 1
Details	542	55	submission	KLHL14	kelch like family member 14
Details	543	54	submission	BMP3	bone morphogenetic protein 3
Details	544	54	submission	EYA3	EYA transcriptional coactivator and phosphatase 3
Details	545	54	submission	TFRC	transferrin receptor
Details	546	54	submission	NSG2	neuronal vesicle trafficking associated 2
Details	547	54	submission	LRIT3	leucine rich repeat, Ig-like and transmembrane domains 3
Details	548	54	submission	CD55	CD55 molecule (Cromer blood group)
Details	549	54	submission	TRIM59	tripartite motif containing 59
Details	550	54	submission	DPY19L2	dpy-19 like 2
Details	551	54	submission	NEUROD4	neuronal differentiation 4
Details	552	54	submission	STK38	serine/threonine kinase 38
Details	553	54	submission	FGD4	FYVE, RhoGEF and PH domain containing 4
Details	554	54	submission	IQGAP1	IQ motif containing GTPase activating protein 1
Details	555	54	submission	HELZ	helicase with zinc finger
Details	556	54	submission	SEL1L	SEL1L, ERAD E3 ligase adaptor subunit
Details	557	54	submission	FAM98B	family with sequence similarity 98 member B
Details	558	54	submission	FCHSD2	FCH and double SH3 domains 2
Details	559	54	submission	NDUFB4	NADH:ubiquinone oxidoreductase subunit B4
Details	560	54	submission	EXTL2	exostosin like glycosyltransferase 2
Details	561	54	submission	CDC7	cell division cycle 7
Details	562	54	submission	MS4A6A	membrane spanning 4-domains A6A

Details	563	54	submission	ERICH1	glutamate rich 1
Details	564	54	submission	ATXN1L	ataxin 1 like
Details	565	53	submission	ACSL4	acyl-CoA synthetase long chain family member 4
Details	566	53	submission	CXorf21	chromosome X open reading frame 21
Details	567	53	submission	CDC27	cell division cycle 27
Details	568	53	submission	CCR3	C-C motif chemokine receptor 3
Details	569	53	submission	PYGO1	pygopus family PHD finger 1
Details	570	53	submission	PCNX1	pecanex 1
Details	571	53	submission	CYB561D1	cytochrome b561 family member D1
Details	572	53	submission	OSCP1	organic solute carrier partner 1
Details	573	53	submission	RIC8A	RIC8 guanine nucleotide exchange factor A
Details	574	53	submission	SSR3	signal sequence receptor subunit 3
Details	575	53	submission	MINDY2	MINDY lysine 48 deubiquitinase 2
Details	576	53	submission	ISG20	interferon stimulated exonuclease gene 20
Details	577	53	submission	KLF8	Kruppel like factor 8
Details	578	53	submission	UVRAG	UV radiation resistance associated
Details	579	53	submission	MGAT4A	alpha-1,3-mannosyl-glycoprotein 4-beta-N-acetylglucosaminyltransferase A
Details	580	53	submission	EXOC6B	exocyst complex component 6B
Details	581	53	submission	RPE	ribulose-5-phosphate-3-epimerase
Details	582	53	submission	PDP2	pyruvate dehyrogenase phosphatase catalytic subunit 2
Details	583	53	submission	APOLD1	apolipoprotein L domain containing 1
Details	584	53	submission	FBXO43	F-box protein 43
Details	585	53	submission	LOC284898	uncharacterized LOC284898
Details	586	53	submission	TMEM161B	transmembrane protein 161B
Details	587	53	submission	SUDS3	SDS3 homolog, SIN3A corepressor complex component
Details	588	53	submission	WDR77	WD repeat domain 77
Details	589	53	submission	ZNF317	zinc finger protein 317
Details	590	53	submission	SNAPC5	small nuclear RNA activating complex polypeptide 5
Details	591	52	submission	FEM1B	fem-1 homolog B
Details	592	52	submission	RMND1	required for meiotic nuclear division 1 homolog
Details	593	52	submission	KIAA2026	KIAA2026
Details	594	52	submission	TPD52L3	TPD52 like 3
Details	595	52	submission	HIPK3	homeodomain interacting protein kinase 3
Details	596	52	submission	CAMK2D	calcium/calmodulin dependent protein kinase II delta
Details	597	52	submission	TFAP2B	transcription factor AP-2 beta
Details	598	52	submission	C1orf61	chromosome 1 open reading frame 61

Details	599	52	submission	KATNBL1	katanin regulatory subunit B1 like 1
Details	600	52	submission	IRF6	interferon regulatory factor 6
Details	601	52	submission	RAB3B	RAB3B, member RAS oncogene family
Details	602	52	submission	SP1	Sp1 transcription factor
Details	603	52	submission	BACE1	beta-secretase 1
Details	604	52	submission	ZNF562	zinc finger protein 562
Details	605	52	submission	LOC100130451	uncharacterized LOC100130451
Details	606	52	submission	CXCL5	C-X-C motif chemokine ligand 5
Details	607	52	submission	EIPR1	EARP complex and GARP complex interacting protein 1
Details	608	51	submission	SMG1	SMG1, nonsense mediated mRNA decay associated PI3K related kinase
Details	609	51	submission	TMEM170A	transmembrane protein 170A
Details	610	51	submission	DCLK2	doublecortin like kinase 2
Details	611	51	submission	C1orf74	chromosome 1 open reading frame 74
Details	612	51	submission	INHBE	inhibin subunit beta E
Details	613	51	submission	DVL3	dishevelled segment polarity protein 3
Details	614	51	submission	ZNF576	zinc finger protein 576
Details	615	51	submission	STEAP2	STEAP2 metalloreductase
Details	616	51	submission	ATP6V1G3	ATPase H+ transporting V1 subunit G3
Details	617	51	submission	PAIP1	poly(A) binding protein interacting protein 1
Details	618	51	submission	COL10A1	collagen type X alpha 1 chain
Details	619	51	submission	FANCL	FA complementation group L
Details	620	51	submission	ZNF559-ZNF177	ZNF559-ZNF177 readthrough
Details	621	51	submission	FABP7	fatty acid binding protein 7
Details	622	51	submission	LYRM2	LYR motif containing 2
Details	623	51	submission	HNRNPU	heterogeneous nuclear ribonucleoprotein U
Details	624	51	submission	CELF2	CUGBP Elav-like family member 2
Details	625	51	submission	SETDB1	SET domain bifurcated histone lysine methyltransferase 1
Details	626	51	submission	KLF3	Kruppel like factor 3
Details	627	51	submission	SYT4	synaptotagmin 4
Details	628	51	submission	HSD11B2	hydroxysteroid 11-beta dehydrogenase 2
Details	629	51	submission	WDR78	WD repeat domain 78
Details	630	51	submission	NRG3	neuregulin 3
Details	631	51	submission	TRIM49	tripartite motif containing 49
Details	632	51	submission	TRIM49C	tripartite motif containing 49C
Details	633	51	submission	RPS6KA5	ribosomal protein S6 kinase A5
Details	634	51	submission	GALNT16	polypeptide N-acetylgalactosaminyltransferase 16

Details	635	51	submission	SPIRE1	spire type actin nucleation factor 1
Details	636	51	submission	TMEM170B	transmembrane protein 170B
Details	637	51	submission	DTWD1	DTW domain containing 1
Details	638	51	submission	PARP11	poly(ADP-ribose) polymerase family member 11
Details	639	51	submission	SFRP1	secreted frizzled related protein 1
Details	640	51	submission	TAOK1	TAO kinase 1
Details	641	51	submission	TRIM49D2	tripartite motif containing 49D2
Details	642	51	submission	FFAR4	free fatty acid receptor 4
Details	643	51	submission	TMEM260	transmembrane protein 260
Details	644	51	submission	KHDC1	KH domain containing 1
Details	645	51	submission	ATL1	atlastin GTPase 1
Details	646	51	submission	YIPF6	Yip1 domain family member 6
Details	647	51	submission	PIK3CG	phosphatidylinositol-4,5-bisphosphate 3-kinase catalytic subunit gamma
Details	648	50	submission	MOB3B	MOB kinase activator 3B
Details	649	50	submission	PPIL6	peptidylprolyl isomerase like 6
Details	650	50	submission	TFDP2	transcription factor Dp-2
Details	651	50	submission	MRE11	MRE11 homolog, double strand break repair nuclease
Details	652	50	submission	ZNF747	zinc finger protein 747
