# Supplementary material for: Ebola Virus Encodes Two microRNAs in Huh7-Infected Cells
Source: Int J Mol Sci. 2022 May 7;23(9):5228. doi: 10.3390/ijms23095228 (PMC9106010; doi:10.3390/ijms23095228)
Supplement: Supplementary file 1 [file ijms-23-05228-s001.zip › Suppl_File_S4_DLA_Constructions_psiCHECKII.pdf]

Ebola virus encodes for two microRNAs in Huh7-infected cells

Idrissa Diallo[1], Zeinab Husseini[1], Sara Guellal[1], Elodie Vion[1], Jeffrey Ho[1], Robert Kozak[2,3], Gary Kobinger[1,2,4] and Patrick Provost[1*]

[1] CHU de Québec Research Center/CHUL Pavilion, 2705 Blvd Laurier, Room T1-65, Quebec, QC G1V 4G2,
Canada, and Department of Microbiology, Infectious Diseases and Immunology, Faculty of Medicine,
Université Laval, Quebec, QC G1V 4G2, Canada;
[2] Special Pathogens Program, National Microbiology Laboratory, Public Health Agency of Canada,
Winnipeg, MB R3B 3M9, Canada;
[3] Department of Laboratory Medicine & Molecular Diagnostics, Division of Microbiology, Sunnybrook Health
Sciences Centre, Toronto, ON M4N 3M5, Canada;
[4] Département de Microbiologie Médicale, Université du Manitoba, Winnipeg, MB R3E 0J9, Canada.
* Correspondence: patrick.provost@crchudequebec.ulaval.ca; Tel.: +1-418-525-4444 (ext. 48842)

File S4.

>NM_030640.3 Homo sapiens dual specificity phosphatase 16 (DUSP16), mRNA

DUSP16 3'UTR-WT with XhoI and NotI RS + *some additional nucleotides to facilitate digestion*

TAAGCA**CTCGAG**ACTTGTGACTTCTATAGACAATTTTTTTTTCTTGTTCACAAAAA
AATTCCCTGTAAATCTGAAATATATGTACATACATATATATTTTTGGAAAATGGAG
CTATGGTGTAAAAGCAACAGGTGGATCAACCCAGTTGTTACTCTCTTAACATCTG
CATTTGAGAGATCAGCTAATACTTCTCTCAACAAAAATGGAAGGGCAGATGCTAG
AATCCCCCCTAGACGGAGGAAAACCATTTTATTCAGTGAATTACACATCCTCTTGT
TCTTAAAAAAGCAAGTGTCTTTGGTGTTGGAGGACAAAATCCCCTACCATTTTCAC
GTTGTGCTACTAAGAGATCTCAAATATTAGTCTTTGTCCGGACCCTTCCATAGTAC
ACCTTAGCGCTGAGACTGAGCCAGCTTGGGGGTCAGGTAGGTAGACCCTGTTAG
GGACAGAGCCTAGTGGTAAATCCAAGAGAAATGATCCTATCCAAAGCTGATTCAC
AAACCCACGCTCACCTGACAGCCGAGGGACACGAGCATCACTCTGCTGGACGGA
CCATTAGGGGCCTTGCCAAGGTCTACCTTAGAGCAAACCCAGTACCTCAGACAGG
AAAGTCGGGGCTTTGACCACTACCATATCTGGTAGCCCATTTTCTAGGCATTGTG
AATAGGTAGGTAGCTAGTCACACTTTTCAGACCAATTCAAACTGTCTATGCACAAA
ATTCCCGTGGGCCTAGATGG**GCGGCCGC*****ATTCGT.***

DUSP16 3’UTR-MUT (transversion mutation) with XhoI and NotI RS + *some additional nucleotides to facilitate digestion*

***TAAGCA*CTCGAG**ACTTGTGACTTCTATAGACAATTTTTTTTTCTTGTGCCCCACA
AAATTCCCTGTAAATCTGAAATATATGTACATACATATATATTTTTGGAAAATGGA
GCTATGGTGTAAAAGCAACAGGTGGATCAACCCAGTTGTTACTCTCTTAACATCT
GCATTTGAGAGATCAGCTAATACTTCTCTCAACAAAAATGGAAGGGCAGATGCTA
GAATCCCCCCTAGACGGAGGAAAACCATTTTATTCAGTGAATTACACATCCTCTTG
TTCTTAAAAAAGCAAGTGTCTTTGGTGTTGGAGGACAAAATCCCCTACCATTTTCA
CGTTGTGCTACTAAGAGATCTCAAATATTAGTCTTTGTCCGGACCCTTCCATAGTA
CACCTTAGCGCTGAGACTGAGCCAGCTTGGGGGTCAGGTAGGTAGACCCTGTTA
GGGACAGAGCCTAGTGGTAAATCCAAGAGAAATGATCCTATCCAAAGCTGATTAA
AACACCCACGCTCACCTGACAGCCGAGGGACACGAGCATCACTCTGCTGGACGG
ACCATTAGGGGCCTTGCCAAGGTCTACCTTAGAGCAAACCCAGTACCTCAGACAG
GAAAGTCGGGGCTTTGACCACTACCATATCTGGTAGCCCATTTTCTAGGCATTGT
GAATAGGTAGGTAGCTAGTCACACTTTTCAGACCAATTCAAACTGTCTATGAAAAC
ACTTCCCGTGGGCCTAGATGG**GCGGCCGC*ATTCGT.***

>NM_003718.5 Homo sapiens cyclin dependent kinase 13 (CDK13), mRNA

CDK13 3’UTR-WT with XhoI and NotI RS + *some additional nucleotides to facilitate digestion*

TAAGCA**CTCGAG**AAAATAAAACACAACCTTTCTCTTGATGCAACAGTTTTATAAA
AAAAAAAATGGTCAACGTTATTTTTGTTTTGTTTTGCAGATTATCAAAGCCTAGCA
AAATGCATTTAAATGAAATAGTGGGTTTTATATGAAAACTATGGGTGGGGTGGGG
AGGGAAAGTAAGTGCCTTAACAGGTAAGCTTAAGGTCTGAAAAAAATAGTTAACT
TTTACCCCCATTTGTCTTTTAAAGGGAATCAATGCATTAAAAAGGCAAGAAACTCT
GAAGTTTCAGGGGCTGCCTGTTCAGTTCCTGTTAGGTCCATTCCCTCTGCTTGTC
ACACTTTCTCCTTTTCTGCCTGTCTTTGGGCTGTGTTGCCTTTCACTACCACCTTCT
CTTGATAGGGGAGTGAGAGCAAAGA**GCGGCCGC*ATTCGT***

CDK13 3’UTR-MUT with XhoI and NotI RS + *some additional nucleotides to facilitate digestion*

TAAGCA**CTCGAG**AAAATAAAACACAACCTTTATATGGCTGCAACAGTTTTATAAA
AAAAAAAATGGTCAACGTTATTTTTGTTTTGTTTTGCAGATTATCAAAGCCTAGCA
AAATGCATTTAAATGAAATAGTGGGTTTTATATGAAAACTATGGGTGGGGTGGGG
AGGGAAAGTAAGTGCCTTAACAGGTAAGCTTAAGGTCTGAAAAAAATAGTTAACT
TTTACCCCCATTTGTCTTTTAAAGGGAATCAATGCATTAAAAAGGCAAGAAACTCT
GAAGTTTCAGGGGCTGCCTGTTCAGTTCCTGTTAGGTCCATTCCCTCTGCTTGTC
ACACTTTCTCCTTTTCTGCCTGTCTTTGGGCTGTGTTGCCTTTCACTACCACCTTC
GCGTTAAAGGGGAGTGAGAGCAAAGA**GCGGCCGC*ATTCGT***
